# Rationalising Heteronuclear Decoupling in Refocussing Applications of Solid‐State NMR Spectroscopy

**DOI:** 10.1002/cphc.201601003

**Published:** 2017-01-23

**Authors:** Ilya Frantsuzov, Suresh K. Vasa, Matthias Ernst, Steven P. Brown, Vadim Zorin, Arno P. M. Kentgens, Paul Hodgkinson

**Affiliations:** ^1^ Department of ChemistryDurham UniversitySouth RoadDurhamDH1 3LEUnited Kingdom; ^2^ Institute for Molecules and MaterialsRadboud UniversityHeyendaalseweg 1356525 EDNijmegenThe Netherlands; ^3^ Laboratory of Physical ChemistryETH ZürichVladimir-Prelog-Weg 28093ZürichSwitzerland; ^4^ Department of PhysicsUniversity of WarwickCoventryCV4 7ALUnited Kingdom; ^5^ Agilent Technologies (UK) Ltd.6 Mead RoadYarntonOxfordshireOX5 1QUUnited Kingdom; ^6^ Mestrelab ResearchS.L Feliciano Barrera 9B—Bajo15706Santiago de CompostelaSpain

**Keywords:** decoupling, magic-angle spinning, nutation, organic solids, solid-state NMR spectroscopy

## Abstract

Factors affecting the performance of ^1^H heteronuclear decoupling sequences for magic‐angle spinning (MAS) NMR spectroscopy of organic solids are explored, as observed by time constants for the decay of nuclear magnetisation under a spin‐echo ($T_{2}^{'}$ ). By using a common protocol over a wide range of experimental conditions, including very high magnetic fields and very high radio‐frequency (RF) nutation rates, decoupling performance is observed to degrade consistently with increasing magnetic field. Inhomogeneity of the RF field is found to have a significant impact on $T_{2}^{'}$ values, with differences of about 20 % observed between probes with different coil geometries. Increasing RF nutation rates dramatically improve robustness with respect to RF offset, but the performance of phase‐modulated sequences degrades at the very high nutation rates achievable in microcoils as a result of RF transients. The insights gained provide better understanding of the factors limiting decoupling performance under different conditions, and the high values of $T_{2}^{'}$ observed (which generally exceed previous literature values) provide reference points for experiments involving spin magnetisation refocussing, such as 2D correlation spectra and measuring small spin couplings.

## 1 Introduction

Effective decoupling of ^1^H nuclear spins is essential for achieving high‐resolution ^13^C and ^15^ n solid‐state NMR spectra from typical organic molecules, and is particularly important for correlation experiments that use J (or scalar) couplings to determine molecular connectivity. Such experiments are central to the use of NMR spectroscopy for establishing molecular structure and dynamics. Moreover, measurement of small J couplings, such as those across N−H⋯ N hydrogen bonds, provides direct information on molecular assembly.^1^ As J couplings are small, relatively long periods (tens of ms) of evolution are required to build up the required spin coherences. Imperfect decoupling of the ^1^H spins leads to significant magnetisation losses during these periods, directly affecting the viability of experiments. For example, the refocussed INADEQUATE experiment^2^ used to assign the ^13^C spectra of testosterone solid forms^3^ required three days, whereas a recent experiment to characterise the organic components of a solid electrolyte interphase^4^ required a 14 day experimental run, despite ^13^C labelling. Isotopic enrichment was also used when probing biopolymers in secondary plant cell walls^5^ and when establishing supramolecular assembly in oxidative polymerisation of aniline^6^ and in rosette nanotubes (using analogous ^15^ n experiments),^7^ whereas specialist dynamic nuclear polarisation techniques have been recently used to obtain correlation spectra of natural abundance samples.^8^, ^9^

The viability of experiments exploiting J couplings in organic molecules is directly related to the rate at which ^13^C magnetisation decays as a result of imperfect decoupling. Although considerable progress has been made in developing approaches to decoupling and understanding how they work,^10^, ^11^, ^12^ there is no comprehensive theory that allows decoupling performance to be quantitatively predicted. Indeed, our earlier work^13^ has shown that *quantitative* reproduction of experimental data through simulation is intrinsically difficult owing to the rapid population of high‐order coherences. Existing experimental studies focus on the important goals of improving spectral linewidths, often through new sequences, including studies of how to choose between sequences.^14^, ^15^, ^16^, ^17^, ^18^, ^19^, ^20^, ^21^, ^22^, ^23^, ^24^, ^25^, ^26^, ^27^ The varied conditions used in these studies, however, make it difficult to establish an overview of the factors determining decoupling performance. Here, we deliberately focus on well‐characterised decoupling sequences under a wide variety of experimental conditions to make more direct and quantitative links between theory and practice. We also concentrate on the regime where the radio‐frequency (RF) nutation rate exceeds the spinning rate. The “low power” regime, where the magic‐angle spinning drives the decoupling, is important for systems that are sensitive to RF heating, such as biological systems. The very different mode of operation,^10^ however, means it is difficult to compare the regimes, and so we focus on the “high power” regime, which is more typical for chemical applications involving organic solids.

As in our earlier study,^13^ the decay time constant under spin‐echo, $T_{2}^{'}$ , is chosen as the primary experimental metric because it is unaffected by inhomogeneous contributions to the spectral linewidth, such as shimming, anisotropic bulk magnetic susceptibility or sample inhomogeneity.^28^, ^29^ Hence $T_{2}^{'}$ continues increasing as the decoupling efficiency increases even though the spectral resolution has plateaued.^30^ It has been noted previously when comparing different decoupling sequences,^21^, ^27^ or different parameters under the same sequence,^31^ that the optimal $T_{2}^{'}$ values tend to vary significantly even though the differences in spectral linewidths at the same conditions are small. So while optimising spectral resolution is relevant for many applications, $T_{2}^{'}$ provides more insight when trying to understand the factors determining decoupling performance, and is directly relevant to the challenging experiments discussed above. The $T_{2}^{'}$ values observed here suggest that J couplings as small as a few Hz are measurable and that J‐based ^13^C correlation experiments should be viable for most systems without the need for isotopic enrichment.

## Techniques and Methods

In common with several previous studies, the methylene group of glycine was used as a model system. The strong dipolar coupling network, both homonuclear and heteronuclear, in methylene groups makes them the hardest type of ^13^C to decouple, ensuring that decoupling that is effective on methylene sites will also be generally effective. Sample data sets acquired by using the methine group of l‐alanine showed similar trends (see Table S1 in the Supporting Information for data sets available), and the conclusions are thus expected to be generally applicable to organic solids.

### Experimental Methods

Experimental measurements of $T_{2}^{'}$ were performed on polycrystalline samples of glycine‐2–^13^C,^15^ n (99 % ^13^C, 98 % ^15^ n) and l‐alanine‐2–^13^C,^15^ n (99 % ^13^C, 98 % ^15^ n) purchased from CortecNet. The glycine sample was confirmed to be α ‐glycine based on the ^13^C carbonyl peak at 176.5 ppm, which is sensitive to polymorphic changes.^32^, ^33^ As expected from the stability range of this form, 5–500 K,^34^ no transformations were observed during experiments.

**Figure 1:**
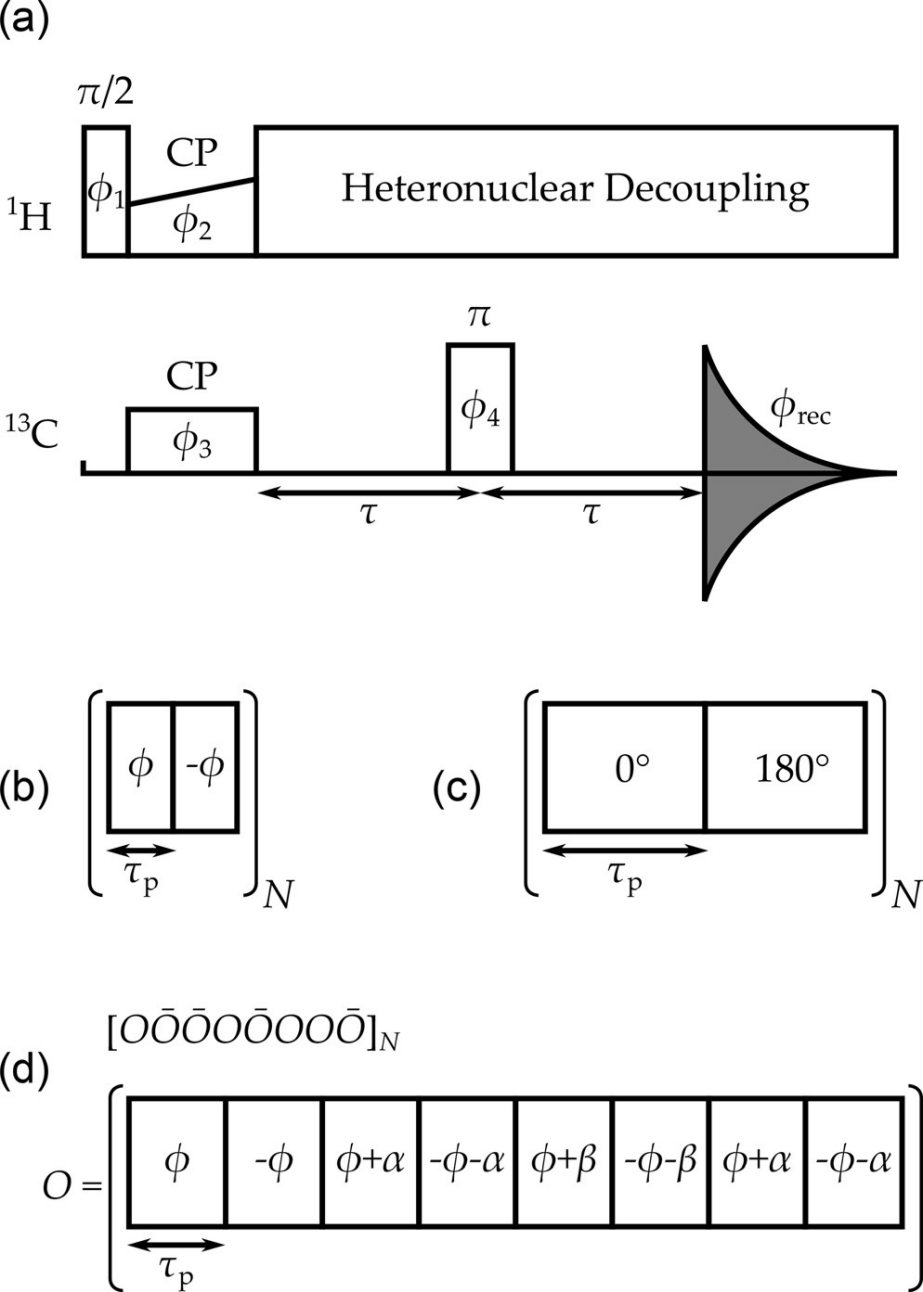
a) Spin‐echo pulse sequence using the same decoupling during the 2τ and acquisition periods. Phases (combining spin‐temperature inversion and selection of refocussed magnetisation): $\phi_{1}=0^{\circ },180^{\circ },0^{\circ },180^{\circ }$ ; $\phi_{2}=\phi_{3}=90^{\circ }$ ; $\phi_{4}=0^{\circ },0^{\circ },90^{\circ },90^{\circ }$ ; $\phi_{\mathrm{rec}}=90^{\circ },270^{\circ },270^{\circ },90^{\circ }$ . b) TPPM heteronuclear decoupling element with phase excursion ϕ and pulse width $\tau_{p}$ . c) XiX heteronuclear decoupling element with pulse width $\tau_{p}$ . d) A single SPINAL block, O , is shown at the bottom, which, together with its counterpart $\bar{O}$ , where all the phases are reversed in sign, are supercycled to make the SPINAL‐64 element, as shown at the top.

Table 1 shows the combinations of hardware used. ^13^C magnetisation was created by using cross‐polarisation (CP), ramped on the ^1^H nutation frequency^35^, ^36^ through the centreband matching condition for $\nu_{r}=12\;\mathrm{kHz}$ , and through the p=+1 zero‐quantum sideband ($\nu_{1}^{H}-\nu_{1}^{C}=p\nu_{r}$ ) for $\nu_{r}=25\;\mathrm{kHz}$ . At $\nu_{r}=62.5\;\mathrm{kHz}$ , CP matching was done on the p=+1 double‐quantum sideband ($\nu_{1}^{H}+\nu_{1}^{C}=p\nu_{r}$ ), as this required much lower RF powers compared with the p=±1 zero‐quantum sideband sidebands commonly employed at slower MAS, but gave comparable signal enhancement.^37^, ^38^ Cross‐polarisation is expected to excite a smaller sample region than direct excitation of the ^13^C magnetisation owing to the effects of RF inhomogeneity.^39^ Tests on a 1.3 mm probe at 25 kHz MAS (hardware configuration 2) showed a larger initial drop in $T_{2}^{'}$ decays when using direct excitation compared with CP, presumably associated with a poorly decoupled sample towards the coil ends, but the long‐term decay and overall fitted $T_{2}^{'}$ values were not significantly different. Although low‐power CP at high MAS rates has been reported to selectively excite the ^13^C spectrum,^40^ such effects were avoided by putting the ^13^C transmitter on the methylene resonance. Relaxation delays were always 4 s, whereas the CP contact times were optimised for each set of experiments, varying in the range 0.8–2.7 ms, as noted in the figure captions. The magnetisation was then measured after a spin‐echo period, τ –π –τ , during which either CW, two‐pulse phase‐modulated (TPPM),^41^ XiX^42^, ^43^ or SPINAL‐64^44^ proton decoupling was applied as shown in Figure 1. As originally defined, the different phase angles in SPINAL‐64 were fixed (10°, 15° and 20°), but have subsequently been optimised, either in the fixed ratio 1:1.5:2 with a single optimisation parameter ϕ , or additionally optimising the angles α and β .^21^, ^45^ Here, a single phase optimisation was used.

**Table 1:** Combinations of probes and consoles.

Configuration	$\nu_{0}^{H}$ [MHz]	Probe	Console
1	300	Bruker 2.5 mm	Varian InfinityPlus
2	500	Bruker 1.3 mm	Varian InfinityPlus
3	600	Bruker 2.5 mm	Bruker Avance II+
4	850	Bruker 2.5 mm	Bruker Avance III
5	850	Bruker 1.3 mm	Bruker Avance III
6	500	Agilent T3 3.2 mm	Agilent DD2
7	500	Agilent BioMAS 3.2 mm	Agilent DD2
8	400	450 μm (i.d.) piggy‐back μMAS^30^, ^47^	Agilent DD2

Generally, the same ^1^H decoupling was used in both spin‐echo and acquisition periods. The only exception was early measurements using the microcoil probe, hardware configuration 8, for which optimised TPPM decoupling at $\nu_{1}=105\;\mathrm{kHz}$ was used during acquisition. As discussed in more detail in Ref. 13 using a fixed decoupling sequence for acquisition gives consistent line‐shapes in the acquired spectra, but significant mismatches between spin‐echo and acquisition decoupling distort fitted $T_{2}^{'}$ values through the orientation dependence of decoupling efficiency.^46^ The ^1^H transmitter frequency was positioned on the maximum of the unresolved ^1^H spectrum at slow MAS. Inversion of ^13^C magnetisation used a π ‐pulse with duration between 5 and 6 μs .

The ^1^H decoupling nutation rate, $\nu_{1}$ , was measured by using the same sequence with a zero spin‐echo period. The initial ^1^H pulse width was incrementally increased in at least 100 steps of approximately $1/(2.5\nu_{1})$ to acquire a ^1^H nutation spectrum and the peak position was taken as the nominal $\nu_{1}$ . TPPM and SPINAL‐64 pulse widths are expressed as a flip angle, $\theta =\tau_{p}\nu_{1}360^{\circ }$ , whereas XiX pulse widths are expressed as a fraction of the rotor period, $\tau_{p}/\tau_{r}$ .

Combinations of MAS and decoupling RF nutation rate were chosen to avoid rotary resonance recoupling conditions at $\nu_{1}=n\nu_{r}$ , where n is an integer. Although the PISSARRO‐5^48^ heteronuclear decoupling sequence was tested, it was found to give very similar parameter maps to XiX (see the Supporting Information), and so we have focussed on the simpler and more readily analysed sequence. This result is not surprising given that PISSARRO was designed to improve performance close to rotary resonance conditions.

Full decay curves were obtained at selected decoupling conditions by incrementing the evolution time, 2τ , linearly in 30–40 steps from zero to approximately twice the maximum expected $T_{2}^{'}$ . The free induction decays were zero‐filled and Fourier transformed (without apodisation) using matNMR.^49^ The decay of the methylene ^13^C peak height as a function of 2τ was fitted to a decaying exponential to obtain $T_{2}^{'}$ using MATLAB.^50^ Where detailed parameter maps as a function of the decoupling sequence parameters were acquired, $T_{2}^{'}$ values were inferred from a pair of experiments at 2τ=0 and $2\tau \approx T_{2,\max}^{'}$ by assuming a mono‐exponential decay of the peak height between these points. As previously discussed in Ref. 13 and reproduced here in the Supporting Information, discrepancies between the $T_{2}^{'}$ values obtained by this quick, but approximate, approach, and those obtained from full decays were corrected by scaling the approximate values to coincide with the more accurate values obtained from full decays. The experiments also provided data for estimating “$T_{2}^{*}$ ”, the effective time constant describing the linewidth, calculated from 1/(πFWHM) , where FWHM is the full width at half‐maximum.

Experiments to assess the dependence of the decoupling performance on magnetic field were performed at ^1^H Larmor frequencies of $\nu_{0}^{H}=300$ , 600 and 850 MHz, at 12 kHz MAS rate and 105 kHz ^1^H nutation rate, using the same 2.5 mm o.d. rotor and similar probe designs. Additional data was collected at $\nu_{0}^{H}=500\;\mathrm{MHz}$ under the same MAS and decoupling nutation rates using hardware configuration 2. For TPPM and SPINAL‐64, full $T_{2}^{'}$ parameter maps were first acquired as a function of both pulse width and phase to locate the positions of optima, then a detailed parameter cross‐section at a fixed pulse phase was recorded through the region of peak decoupling, which was $\phi =6^{\circ }$ for both sequences under these conditions. The XiX performance was first characterised over a wide range of pulse lengths, and then in more detail over the region of peak decoupling to ensure the narrow optima were well defined.

Decoupling experiments using very high RF decoupling fields, beyond the reach of commercially available probes, were performed using a piggyback μMAS design equipped with a 450 μm inner diameter coil, as described in Ref. 30, 47.

Experiments to assess the influence of RF field inhomogeneities on decoupling were performed using two 3.2 mm MAS probes with different coil geometries: an Agilent T3 MAS probe with standard solenoid coil geometry, and an Agilent BioMAS probe, whose scroll coil geometry exhibits significantly better $B_{1}$ homogeneity. The same sample rotor, MAS rate, and spectrometer equipment were used in both sets of experiments, and care was taken to adjust the power levels for each probe such that the peak nutation frequencies were the same.

### Simulations

Numerical simulations of the decay of ^13^C magnetisation in the presence of ^1^H decoupling were performed using pNMRsim,^51^ as described in Ref. 13 and reproduced here in the Supporting Information. Spin systems are labelled as CH_*n*_, with n indicating the number of protons coupled to the central carbon atom. The decays were fitted to a single‐exponential function to derive computed dephasing time constants, $T_{2}^{c}$ , describing the loss of the ^13^C single‐quantum coherence. Note that spin‐echoes are not included in the simulation as there are no inhomogeneous components of the decay to refocus, and computations of $T_{2}^{'}$ are much more sensitive to finite‐sized spin systems.^13^ The simulations incorporate effects of RF inhomogeneity through nutation spectra acquired using the same equipment and experimental conditions. As described in more detail in the Supporting Information, simulations were performed for a set of 15–20 RF nutation rates chosen to correspond to equal areas of the nutation profile, and the results summed.

## 2 Results

Determining the optimal decoupling parameters across a range of experimental set‐ups involved acquiring a large number of detailed parameter maps for both $T_{2}^{*}$ and $T_{2}^{'}$ . As these were acquired under a uniform set of conditions, these data should be a useful resource for further study. A summary of the data sets available is given in the Supporting Information.

An initial comparison of the data highlights that ease of optimisation varies significantly between sequences and experimental conditions. These aspects of optimisation have previously been discussed for peak height^24^ and $T_{2}^{'}$ .^21^ Optimisation becomes more difficult as the ratio of the RF nutation rate to MAS frequency reduces, for example, going from $\nu_{r}=25$ to 62.5 kHz under $\nu_{1}=170$ kHz decoupling. As illustrated by Figures S9–S11 in the Supporting Information, the parameter maps became fragmented by multiple destructive resonance conditions, requiring finer parameter grid increments. For TPPM and SPINAL‐64, the optimum pulse phase moved away from the commonly prescribed $\phi =7^{\circ }$ and $\phi =6^{\circ }$ ,^45^ respectively, necessitating the optimisation of both pulse width and phase. SPINAL‐64 optima are especially narrow, likely owing to the increased number of resonance conditions due to the longer cycle time of the sequence. The XiX parameter map underwent relatively few changes, making it easier to optimise at high MAS frequencies compared with TPPM or SPINAL‐64.

As has been previously observed, the local minima and maxima across a $T_{2}^{*}$ parameter map qualitatively correspond with those of the $T_{2}^{'}$ map.^21^ The $T_{2}^{'}$ optima were, however, generally narrower, and optima with similar $T_{2}^{*}$ values tended to have different relative $T_{2}^{'}$ values. In other words, a $T_{2}^{*}$ map cannot be relied upon to provide the best sequence parameters for $T_{2}^{'}$ .

### 2.1 Decoupling Transmitter Offset

**Figure 2:**
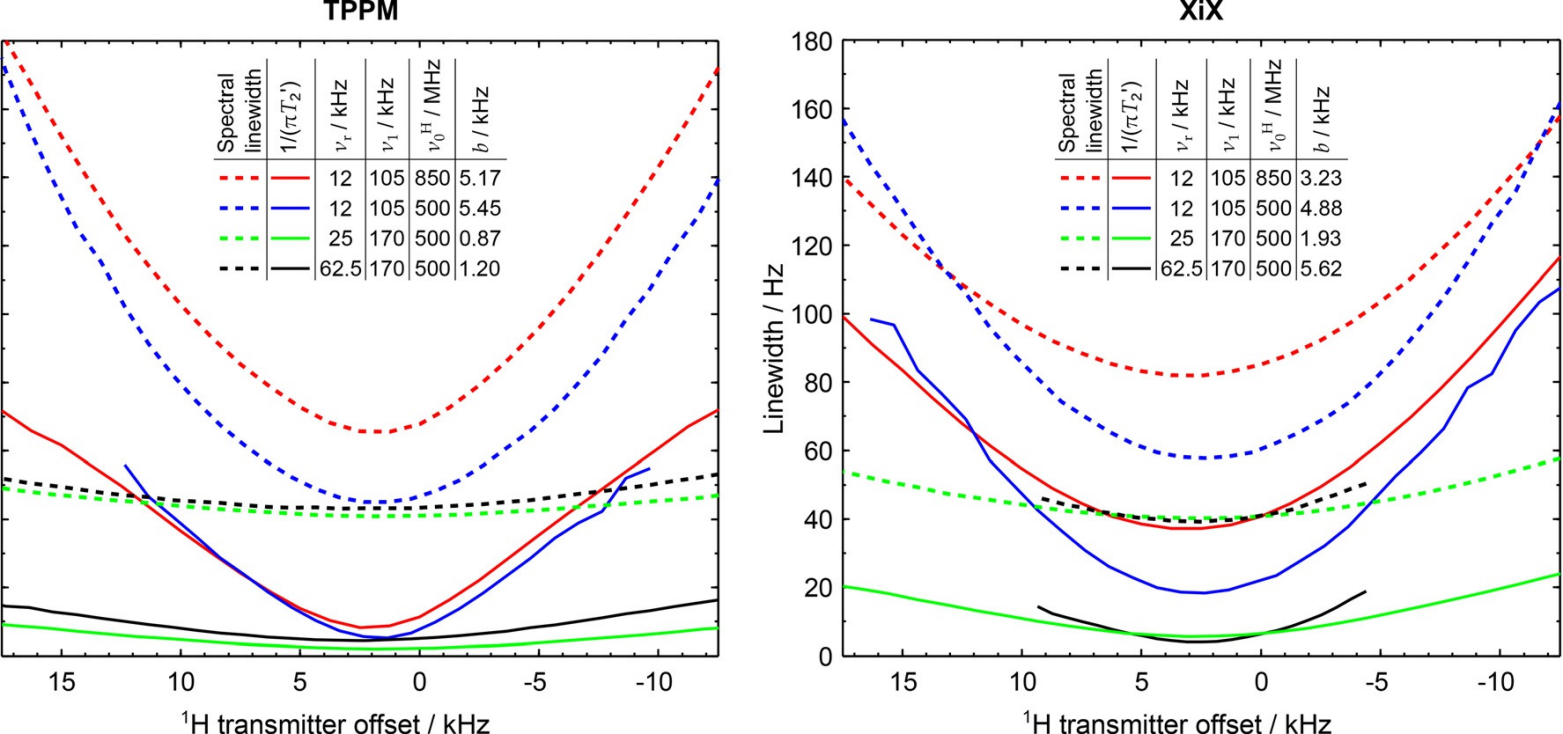
^1^H transmitter offset dependence for optimised TPPM and XiX decoupling under various experimental conditions. The spectral linewidth was measured as the FWHM of the peak. For data at $\nu_{0}^{H}=850\;\mathrm{MHz}$ , hardware configuration 4 was used with a CP contact time of 1.8 ms (see Table 1). For data at $\nu_{0}^{H}=500\;\mathrm{MHz}$ , hardware configuration 2 was used with CP contact times of 1.2, 1.2 and 1.5 ms at $\nu_{r}=12,25$ and 62.5kHz respectively. The b values refer to fits of the spectral linewidths as a function of transmitter offset, Δ , to $\mathrm{LW}={\mathrm{LW}}_{\min}+b{\left(\left(\Delta -\Delta_{\min}\right)/\nu_{1}\right)}^{2}$ .

The detrimental effects of off‐resonance irradiation on decoupling efficiency are well‐established for spectral linewidth,^11^, ^19^, ^22^, ^25^, ^52^ but to a lesser degree for $T_{2}^{'}$ .^53^ It was found that the optimal pulse width (and phase) did not change significantly over a range of ^1^H offsets ±10kHz about the optimum, thus the offset dependence could be determined independently using the same optimal sequence parameters. Figure 2 shows that the characteristic ‘width’ of the offset dependencies is similar between $1/(\pi T_{2}^{*})$ and $1/(\pi T_{2}^{'})$ for TPPM across a range of experimental parameters. CW and SPINAL‐64 (see Figure S5 in the Supporting Information) exhibit the same behaviour. With the exception of XiX decoupling, an increase in the $B_{0}$ field is accompanied by a consistent increase in linewidth for both $1/\pi T_{2}^{*}$ and $1/\pi T_{2}^{'}$ , that is, a vertical offset. XiX has a more complex offset dependence, being more broadband at larger $B_{0}$ .

The dependence of ^13^C linewidths on transmitter offset under CW decoupling was shown to be described by a parabola, and was justified theoretically by VanderHart et al.,^54^ following Mehring.^55^ It is not clear, however, that more complex sequences share this dependence, and indeed some sequences have been specifically optimised to be robust with respect to offset variations.^56^ Figure 2 shows that, although the dependence of spectral linewidth on offset for TPPM decoupling is close to parabolic at modest RF nutation rates, it is clearly not for $1/(\pi T_{2}^{'})$ under the same conditions. The b parameter shown in Figure 2 measures the steepness of the parabolic curve fitted to the offset dependence of the spectral linewidth. From the expressions given by VanderHart, this parameter might be expected to be a fixed function of the NMR parameters, independent of RF nutation rate, but this is clearly not the case, even for CW decoupling. In particular, the robustness with respect to the offset is significantly improved at increased nutation rates, as measured by the decrease in the b parameter. The $1/(\pi T_{2}^{'})$ curves do not fit well to simple parabolas, but show qualitatively similar trends. Although these observations are interesting and worth further investigation, they are not directly relevant to the complex interplay of experimental conditions and spin system dynamics at the heart of the decoupling problem. The following results assume that the ^1^H transmitter offset is close to the optimal conditions.

### 2.2 $B_{0}$ Field Dependence of $T_{2}^{'}$

**Figure 3:**
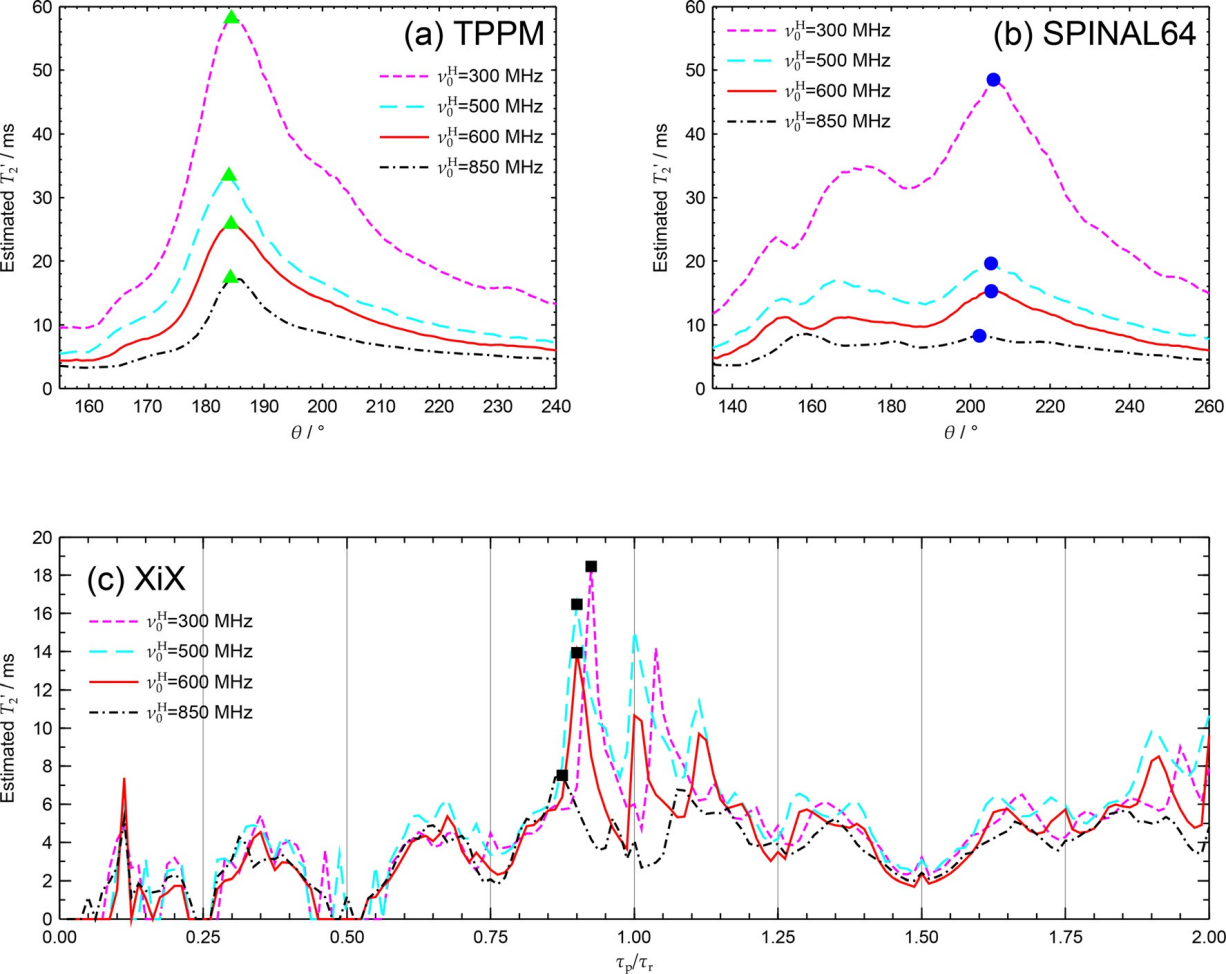
Experimental $T_{2}^{'}$ across a) TPPM, $\phi =6^{\circ }$ , b) SPINAL‐64, $\phi =6^{\circ }$ and c) XiX parameter map cross‐sections at $\nu_{0}^{H}=300\;\mathrm{MHz}$ (magenta), 500 MHz (cyan), 600 MHz (red) and 850 MHz (black). Datasets acquired using $\nu_{r}=12\;\mathrm{kHz}$ and $\nu_{1}=105\;\mathrm{kHz}$ (XiX used $\nu_{r}=11.905\;\mathrm{kHz}$ to ensure synchronisation of pulse width increments with the MAS period). The $2<\tau_{p}/\tau_{r}\leq 8$ region of the XiX parameter maps was relatively featureless and is omitted for clarity. Hardware configurations 1–4 were used, with CP contact times of 2.7, 1.2, 2.7 and 1.8 ms respectively (see Table 1). Small mis‐calibrations of the $\nu_{1}$ by 1 and 4 kHz for the 600 and 850 MHz datasets respectively have been taken into account in (a) and (b) by adjusting the calculated tip‐angle, θ . Peak decoupling points marked by triangles, circles and squares are shown as a function of 1/$\nu_{0}^{H}$ in Figure 4.

**Figure 4:**
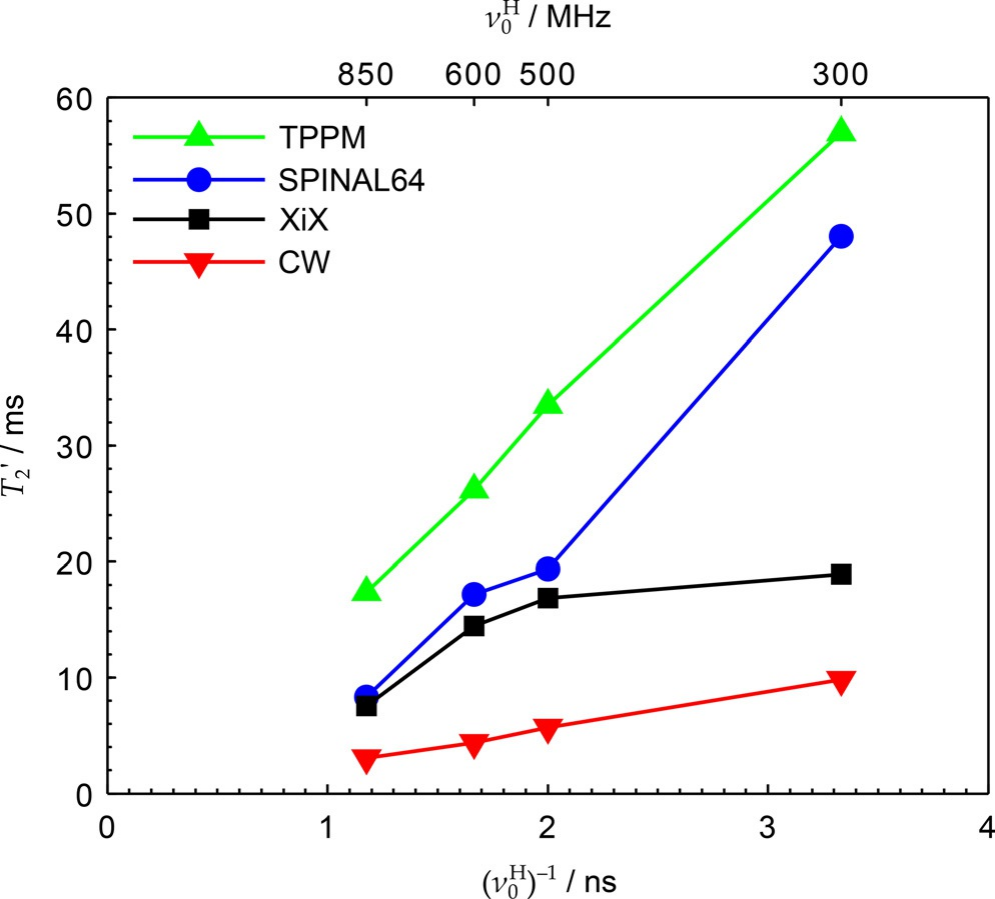
Experimental $T_{2}^{'}$ as a function of 1/$\nu_{0}^{H}$ under optimised TPPM, SPINAL‐64 and XiX decoupling, corresponding to the points marked in Figure 3, as well as under CW. Datasets acquired using $\nu_{r}=12\;\mathrm{kHz}$ and $\nu_{1}=105\;\mathrm{kHz}$ (XiX used $\nu_{r}=11.905\;\mathrm{kHz}$ to ensure synchronisation of pulse width increments with the MAS period). Hardware configurations 1–4 were used, with CP contact times of 2.7, 1.2, 2.7 and 1.8 ms respectively (see Table 1). Uncertainties in the fitted values of $T_{2}^{'}$ were of the order of the marker sizes or smaller.

Parameter maps of decoupling performance at moderate MAS and ^1^H nutation rates (12 kHz and 105 kHz respectively) were acquired at several magnetic fields. Figure 3 shows characteristic sections of these maps for the four $B_{0}$ fields studied. The decoupling optima marked for each decoupling sequence in Figure 3 are collated in Figure 4 as a function of Larmor period, $1/\nu_{0}^{H}$ . The trend in Figure 3 a and Figure 4 of shortening $T_{2}^{'}$ values with increasing $B_{0}$ for CW and TPPM decoupling is consistent with their decoupling performance being dominated by second‐order cross‐terms between the heteronuclear dipolar couplings and ^1^H chemical shift anisotropy (CSA) tensor, which increase proportionately with $B_{0}$ .^57^, ^58^, ^59^ The relative complexity of SPINAL‐64 has hindered its theoretical analysis, but its mode of operation is assumed to be essentially the same as TPPM and so might be expected to have a similar $B_{0}$ dependence. Although the detrimental effects of increasing $B_{0}$ upon $T_{2}^{'}$ can be inferred from previously published data^21^ on glycine, and poorer values of $T_{2}^{'}$ at 600 MHz compared with 300 MHz have been noted for XiX and RS‐HEPT decoupling,^53^ the field dependence has not been explicitly explored. Figures S6 and S7 in the Supporting Information collate results from Ref. 21 together with ours. Note that the apparent extrapolation of the data points for SPINAL‐64 in Figure 4 towards negative $T_{2}^{'}$ values in the limit of infinite magnetic field are likely to be an artefact of increasing off‐resonance effects at higher field; this behaviour is not borne out in the wider collated data of Figure S6. Moreover, unlike TPPM, the SPINAL‐64 parameter map, Figure 3 b, changes shape with $B_{0}$ , and so it may be more difficult to observe consistent trends for more complex sequences. This may explain, for example, why SPINAL‐64 appeared to perform better at 700 MHz compared with 500 MHz in a previous study,^21^ whereas all the other results show the opposite trend.

The linewidth under XiX decoupling, and related sequences such as PISSARRO‐5, is dominated by proximity to resonance conditions as well as second‐order cross‐terms between the heteronuclear and homonuclear dipolar couplings,^60^ and so is not expected to show a strong magnetic field dependence. This is largely confirmed in Figure 3 c, where significant portions of the parameter space have very similar $T_{2}^{'}$ values for all four $B_{0}$ fields, although the peak performance at high field is measurably poorer. The position of the XiX optimum is very sensitive to $\nu_{1}$ under this combination of $\nu_{r}$ and $\nu_{1}$ , in the same way as for low‐power XiX decoupling.^61^ This is evident in Figure 3 c for the decoupling optima at $\tau_{p}/\nu_{1}=8$ , corresponding to $\tau_{p}/\tau_{r}\approx 0.91$ ; owing to their proximity to destructive resonances, the XiX peak $T_{2}^{'}$ values are much more sensitive to $\nu_{1}$ mis‐adjustments than TPPM or SPINAL‐64. Therefore, the peak XiX $T_{2}^{'}$ values may not be robust, especially at $\nu_{0}^{H}=850\;\mathrm{MHz}$ , where the RF inhomogeneity is noticeable poorer (Figure S4 in the Supporting Information). The consensus of these and previous results is that *achievable* XiX performance does decrease as the magnetic field increases.

**Figure 5:**
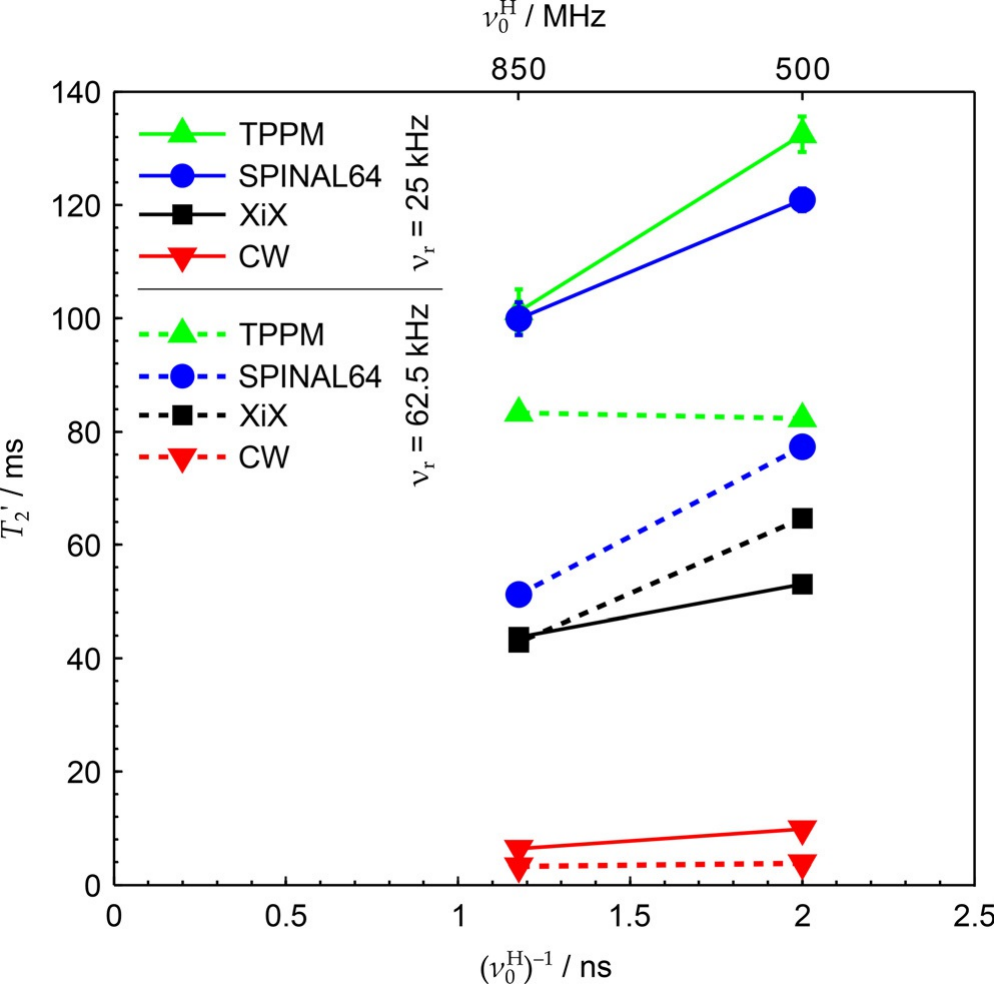
Experimental $T_{2}^{'}$ as a function of 1/$\nu_{0}^{H}$ and $\nu_{r}$ under CW and optimised TPPM, SPINAL‐64 and XiX decoupling under faster spinning conditions than those of Figure 4. Datasets acquired using $\nu_{1}=170\;\mathrm{kHz}$ . Hardware configurations 2 and 5 were used (see Table 1). For $\nu_{r}=25\;\mathrm{kHz}$ , CP contact times were 1.2 and 2.5 ms for configurations 2 and 5 respectively. For $\nu_{r}=62.5\;\mathrm{kHz}$ , CP contact times were 1.5 and 1.2 ms for configurations 2 and 5 respectively.

Figure 5 collates the optimal $T_{2}^{'}$ values at two magnetic fields (corresponding to $\nu_{0}^{H}=500$ and 850 MHz) and two MAS frequencies ($\nu_{r}=25$ and 62.5 kHz) for which complete data sets were obtained for all the decoupling sequences used. It can be seen that the trends of decreasing $T_{2}^{'}$ with $B_{0}$ at $\nu_{r}=25$ kHz (solid lines) are similar to those at $\nu_{r}=12$ kHz in Figure 4—that is, XiX is not as dependent on $B_{0}$ as the other sequences. However, under fast MAS (dashed lines), when the two frequencies, $\nu_{r}$ and $\nu_{1}$ , become more comparable, the direct impact of $B_{0}$ on $T_{2}^{'}$ is less clear. This can be attributed to the increased significance of resonance conditions on the parameter space, complicating interpretations of $T_{2}^{'}$ at optima based on a single dominant mechanism by making the optima narrow and very sensitive to small changes in $\nu_{r}$ and $\nu_{1}$ (Figures S9–S11 in the Supporting Information). Unlike the other sequences considered, the XiX $T_{2}^{'}$ either improves or stays unchanged with increasing MAS rate.

These results complement previous studies of decoupling performance as a function of $\nu_{r}$ for slower spinning frequencies,^27^ where $T_{2}^{'}$ values for the TPPM and SPINAL‐64 decoupling sequences were observed to *increase* up to 20 kHz MAS before falling off, whereas the performance of XiX and CW systematically increased and decreased, respectively, with fast spinning (Figure S8 in the Supporting Information). Although the qualitative picture that emerges is consistent, it is worth noting the actual values of $T_{2}^{'}$ vary markedly between studies (with the exception of simple CW decoupling). The dependence of $T_{2}^{'}$ values on the optimisation protocol, and potentially, hardware details, illustrates the difficulty of drawing conclusions based on individual studies.

### 2.3 $B_{1}$ Field Inhomogeneity

The effects of $B_{1}$ field inhomogeneities are potentially significant for phase‐modulated decoupling sequences, such as TPPM, where the optimum pulse length is strongly dependent on the ^1^H nutation frequency.^41^, ^62^ Most of the literature focus has been on homonuclear decoupling, where different $B_{1}$ fields across a sample produce a distribution of scaling factors, dramatically degrading resolution,^63^ although some heteronuclear decoupling sequences have been expressly designed to be more robust with respect to differences in $B_{1}$ , such as SDROOPY^56^ and SW_*f*_‐TPPM.^52^, ^64^ The influence of $B_{1}$ inhomo‐ geneity on $T_{2}^{'}$ values has, however, not been explicitly investigated.

**Figure 6:**
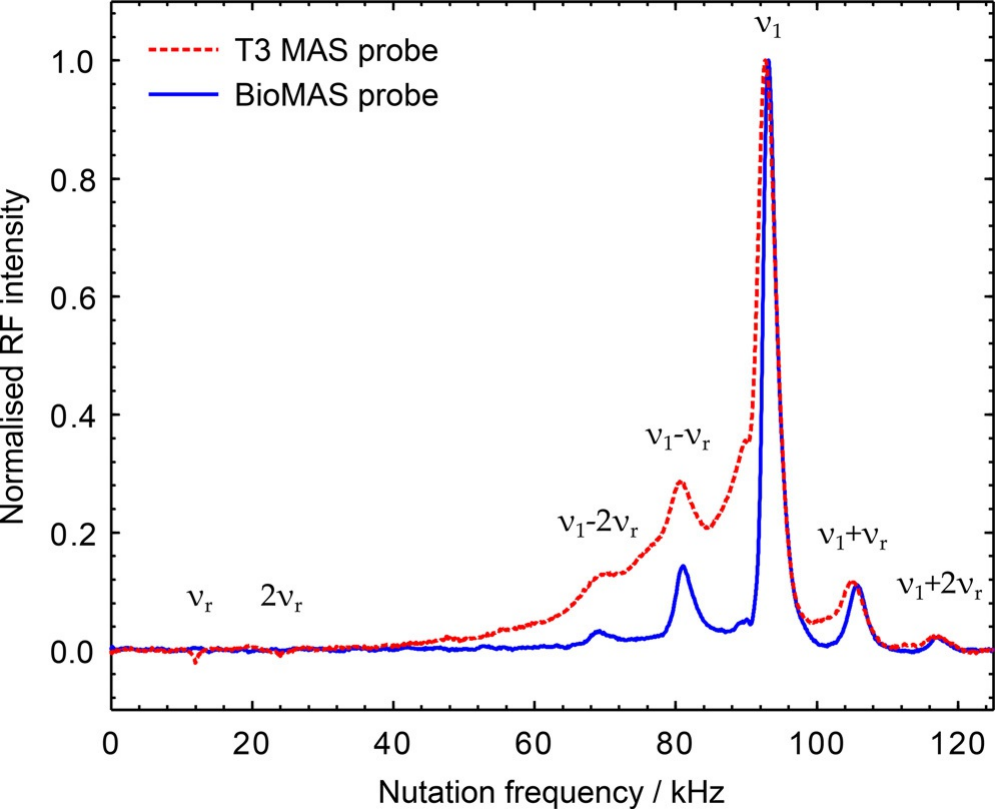
^1^H nutation spectra measured through ^13^C using T3 MAS and BioMAS probes, hardware configurations 6 and 7 respectively (see Table 1). Peak nutation frequencies are at $\nu_{1}=93\;\mathrm{kHz}$ . Spectra acquired at $\nu_{r}=12\;\mathrm{kHz}$ and $\nu_{0}^{H}=500\;\mathrm{MHz}$ using CP conditions with 1.2 ms contact time.

Figure 6 shows ^1^H nutation spectra measured through ^13^C for two 3.2 mm MAS probes with different coil geometries. The measured nutation spectrum of the T3 probe (conventional solenoid coil) clearly shows a peak at $\nu_{1}=93\;\mathrm{kHz}$ and a long tail of much‐reduced RF, which is known from $B_{1}$ inhomogeneity imaging experiments (on a Bruker 4 mm probe) to originate from the sample at the ends of the rotor.^65^ The nutation spectrum of the BioMAS probe (scroll coil geometry) is much narrower, indicating a more homogeneous $B_{1}$ field across the sample. Owing to the additional effects of CP selectivity, determined by the quality of RF matching between the two channels,^39^ these nutation spectra are narrower than ones acquired directly on the proton signal (especially for the T3 probe), but are a better representation of RF experienced by the sample visible in ^13^C CP/MAS spectra. The nutation spectra show additional peaks appearing at frequencies $n\nu_{r}$ and $\nu_{1}\pm n\nu_{r}$ , where n is an integer and $\nu_{r}=12\;\mathrm{kHz}$ . These features were observed with other combinations of probes and experimental conditions used and are likely to result from a time‐dependence of the magnitude and direction of the effective field owing to anisotropic interactions, which are modulated by the MAS frequency.^66^

**Figure 7:**
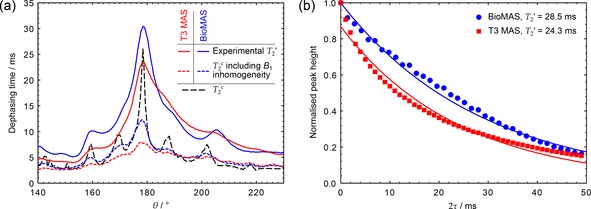
a) Experimental $T_{2}^{'}$ and simulated $T_{2}^{c}$ across TPPM parameter map cross‐sections at $\phi =5^{\circ }$ , using T3 MAS (red) and BioMAS probes (blue), hardware configurations 6 and 7 respectively (see Table 1). Datasets were acquired using $\nu_{r}=12\;\mathrm{kHz}$ , $\nu_{1}=93\;\mathrm{kHz}$ and $\nu_{0}^{H}=500\;\mathrm{MHz}$ , with a CP contact time of 2 ms. A small mis‐calibration of the $\nu_{1}$ by 2 kHz for the T3 probe dataset has been taken into account when calculating the tip‐angle, θ . b) Experimental $T_{2}^{'}$ decay curves under optimal TPPM decoupling, corresponding to the peaks in (a). Solid lines represent fits to a mono‐exponential decay, with the decay constant shown in the legend.

The TPPM parameter space was characterised at 12 kHz MAS and $\nu_{1}=93\;\mathrm{kHz}$ in terms of both pulse duration and phase, and a detailed parameter map cross‐section then recorded at the optimum phase (Figure 7 a). The results clearly show a broadening effect of $B_{1}$ inhomogeneity on the shape of the parameter map as the sample experiences a wider distribution of pulse lengths. There is a corresponding effect on $T_{2}^{'}$ , as seen in Figure 7 b; $B_{1}$ inhomogeneity leads to a distribution of $T_{2}^{'}$ values across the sample and a significant multi‐exponential character in the $T_{2}^{'}$ decays.

The dashed lines in Figure 7 a show fitted time constants, $T_{2}^{c}$ , of simulations of the decay of ^13^C magnetisation in a CH_7_ spin system with and without the effects of $B_{1}$ inhomogeneity, as described in the Simulations section above and in more detail in Section 4 of the Supporting Information. The RF inhomogeneity profiles were obtained from the nutation spectra in Figure 6. Although the calculated $T_{2}^{c}$ values and experimental $T_{2}^{'}$ values are not directly comparable, the simulations show the same qualitative trends and confirm that $B_{1}$ inhomogeneity has a significant impact on decoupling performance.

**Figure 8:**
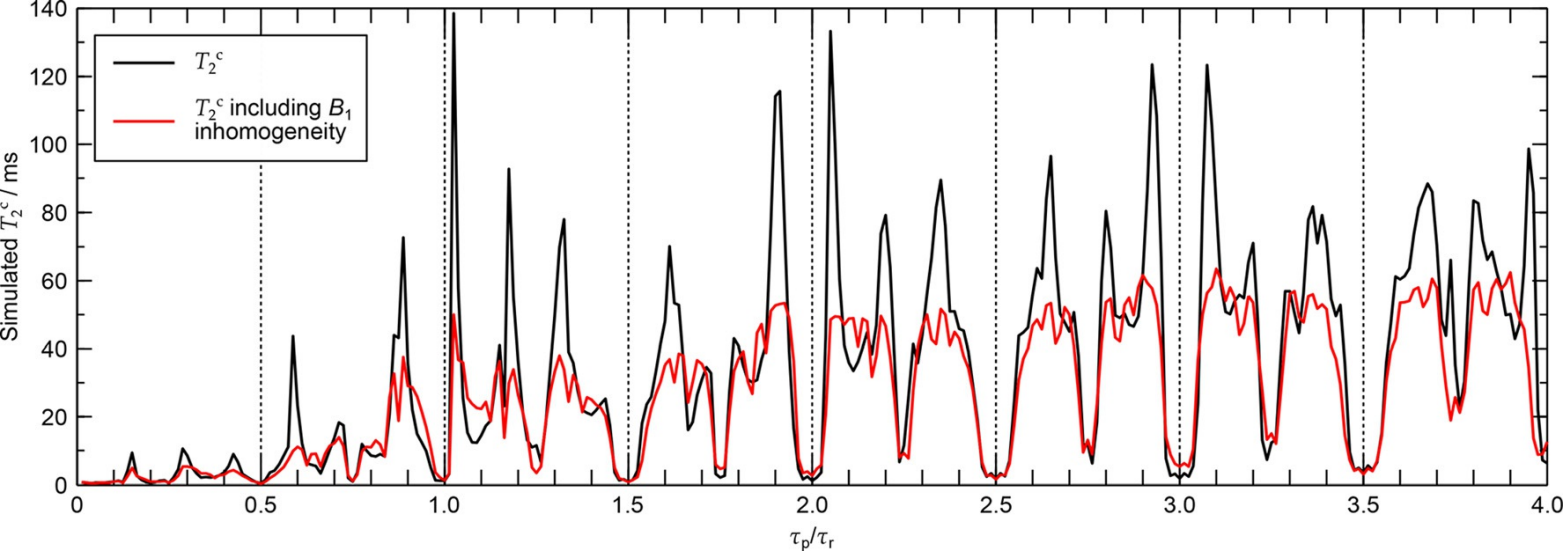
Simulated $T_{2}^{c}$ with and without RF inhomogeneity under XiX decoupling at $\nu_{r}=25\;\mathrm{kHz}$ , $\nu_{1}=170\;\mathrm{kHz}$ and $\nu_{0}^{H}=500\;\mathrm{MHz}$ . RF inhomogeneity was incorporated using 22 points along the T3 nutation spectrum of Figure 6.

Sequences such as XiX, the timings of which are expressed relative to the MAS period rather than in terms of a nutation angle, might be expected to be relatively robust with respect to $B_{1}$ homogeneities. $T_{2}^{c}$ simulations using a CH_8_ spin‐system at $\nu_{r}=25\;\mathrm{kHz}$ , $\nu_{1}=170\;\mathrm{kHz}$ shown in Figure 8 demonstrate, however, that XiX global optima are also quite sensitive to $B_{1}$ homogeneities, with a reduction of $T_{2}^{c}$ by approximately 50 % for typical probe homogeneity profiles. Also, the strong dependence of XiX optima on $\nu_{1}$ calibration under some combinations of $\nu_{r}$ and $\nu_{1}$ , as evident from the experimental results in Figure 3 c at low $\nu_{r}$ and other investigations at high $\nu_{r}$ ,^61^ imply that $B_{1}$ homogeneity is expected to reduce peak decoupling performance in those cases too.

### 2.4 Pulse Transients

The deleterious effects of transient variations in the RF experienced by the sample, associated with changes of the driving amplitude or phase, have been appreciated since the early days of pulse NMR.^67^ Such effects are known to be significant for homonuclear decoupling sequences; Ref. 68 for example, analyses how pulse imperfections influence the effective resolution of windowed PMLG decoupling. Other experiments have been shown to be relatively robust with respect to RF transients,^69^ but, to the best of our knowledge, there is no published work on the effects of RF transients on heteronuclear decoupling.

RF transients can be measured experimentally by pick‐up coils placed close to the NMR sample. At least in the case of the dipolar recoupling experiment considered by Carravetta et al.,^69^ these were shown to fit well both to exact electronic simulations of model tuned coil circuits and to simple mathematical models of the time‐dependence of the RF phase and amplitude. The model described by Equations (10)–(14) of Ref. 69 can be usefully simplified for phase‐modulated sequences (i.e. constant driving RF amplitude). The resulting x (in‐phase) and y (quadrature) components of the magnetic field, $B_{1}\left(t\right)$ , following a phase change at t=0 are [Eq. (1)]:

(1) $$\left.\begin{array}{cc} B_{x}\left(t\right)\; & =B_{1}+B_{1}e^{-\lambda_{\mathrm{trans}}t}\left[\left(1-\cos\Delta \phi \right)+\lambda_{Q}t\sin\Delta \phi \right]\;\\ B_{y}\left(t\right)\; & =B_{1}e^{-\lambda_{\mathrm{trans}}t}\left[-\sin\Delta \phi +\lambda_{Q}t\left(1-\cos\Delta \phi \right)\right]\; \end{array}\;\;\right\}\;t>0$$

where $B_{1}=2\pi \nu_{1}/\gamma$ is the amplitude of the driving RF, and Δϕ is the phase change relative to the initial x phase. $\lambda_{\mathrm{trans}}$ is the rate constant for the transient response of the tuned circuit, which is largely determined by the Q of the probe, and $\lambda_{Q}$ parameterises the amplitude of the quadrature component of the transient response. Although $\lambda_{\mathrm{trans}}$ is essentially fixed by the probe, $\lambda_{Q}$ is largely determined by the mismatch between the frequency of the driving RF and the resonant frequency of the tuned circuit. $B_{x}\left(t\right)$ and $B_{y}\left(t\right)$ are readily converted to an overall RF amplitude and an instantaneous phase as a function of time. Illustrative examples of RF transient profiles can be found in recent literature reports.^69^, ^70^, ^71^, ^72^ Although active compensation of amplitude and phase transients has been implemented for recoupling experiments with promising effects on reproducibility and stability,^72^ it is not clear whether heteronuclear spin decoupling could be similarly improved. Pulse transients will change the effective field and the Fourier coefficients characterising a decoupling sequence, thereby affecting the residual coupling terms and resonance conditions. It is, therefore, difficult to determine a priori whether compensation of the transient response would improve the performance of a given sequence. Simulations are employed here in lieu of a detailed theoretical description.

The transient response is modelled in the simulations by dividing the evolution into short time steps, $\tau_{\mathrm{step}}$ , typically of $1/2\lambda_{\mathrm{trans}}$ . Calculating propagators for each $\tau_{\mathrm{step}}$ making up an individual pulse would be extremely time‐consuming, and so the response is only modelled over the first 5 to 6 time constants (i.e. $t\leq 6/\lambda_{\mathrm{trans}}$ ), and the set phase and amplitude used for the remaining pulse duration. The convergence of the free induction decay (FID) with respect to both parameters was checked on a case‐by‐case basis. The pulses were always sufficiently long compared with $6/\lambda_{\mathrm{trans}}$ so that overlap of the transient responses did not occur. Using a low‐Q pick‐up coil, values of $\lambda_{\mathrm{trans}}=4\;{\mu s}^{-1}$ and $\lambda_{Q}=0.8\;{\mu s}^{-1}$ were obtained by fitting the experimental transient response of a 2.5 mm 400 MHz DR Bruker probe to a $\phi =90^{\circ },\;-90^{\circ }$ pulse pair.

**Figure 9:**
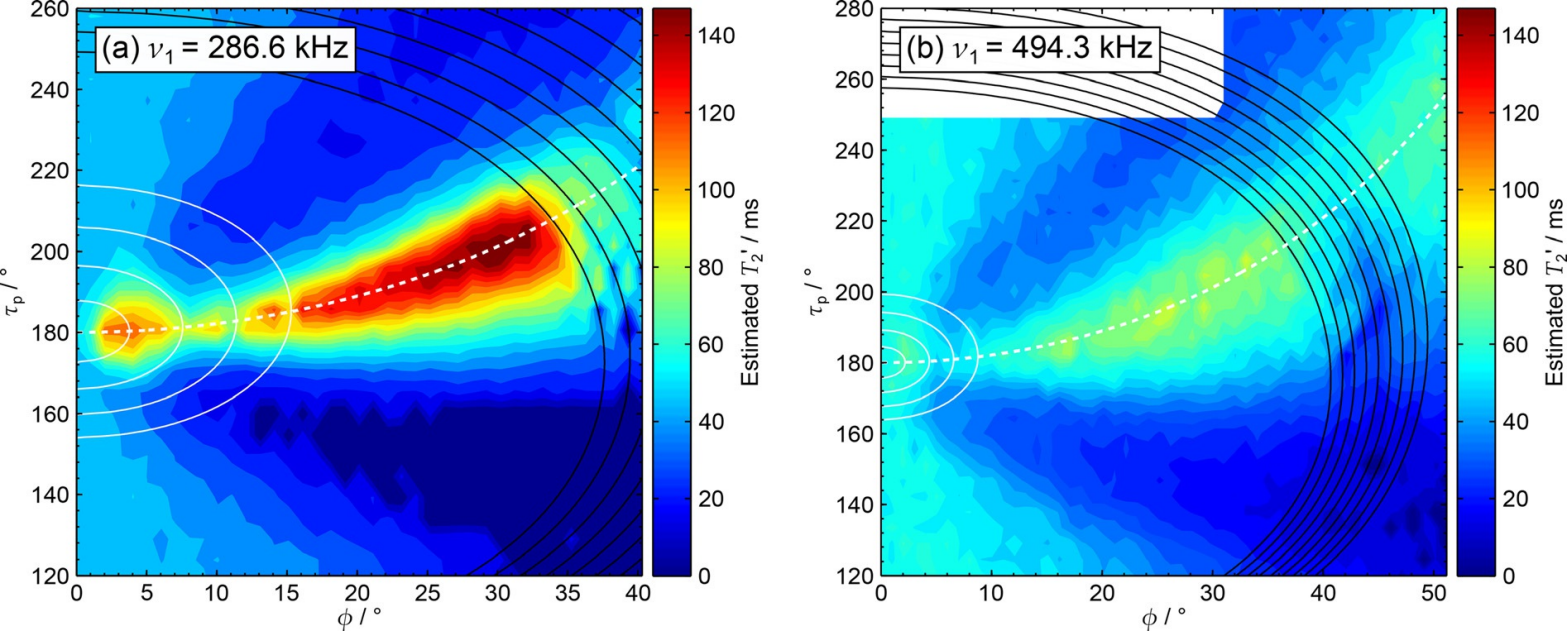
Experimental $T_{2}^{'}$ under TPPM decoupling at $\nu_{r}=12\;\mathrm{kHz}$ , $\nu_{0}^{H}=400\;\mathrm{MHz}$ and a) $\nu_{1}=286.6\;\mathrm{kHz}$ , b) $\nu_{1}=494.3\;\mathrm{kHz}$ . The solid lines represent recoupling resonance conditions for heteronuclear interactions (black) and purely homonuclear interactions (white) as described in Ref. 59. Decoupling optima lie along the dashed white line, $\tau_{p}^{180}/cos\left(\phi /1.1289\right)$ . Hardware configuration 8 was used with a CP contact time of 2 ms (see Table 1). Note that data was not acquired in the white region of (b).

The effects of transients are expected to be larger at the high nutation rates routinely available when using a microcoil MAS probe.^73^ This was investigated for TPPM and SPINAL‐64 decoupling at $\nu_{r}=12\;\mathrm{kHz}$ and a range of nutation frequencies up to $\nu_{1}=500$ kHz. Under conditions of $\nu_{1}\gg \nu_{r}$ , the TPPM pulse width and phase were easily optimised, as shown by the relative sparsity of the resonance conditions across the parameter maps in Figure 9 compared with those at lower nutation frequencies (Figure S9 in the Supporting Information). In both experiment and simulation, optimal decoupling was found to lie along the line $\tau_{p}^{180}/cos\left(\phi /1.1289\right)$ , close to the $\tau_{p}^{180}/cos\left(\phi \right)$ predicted by Floquet analysis.^52^, ^59^

**Figure 10:**
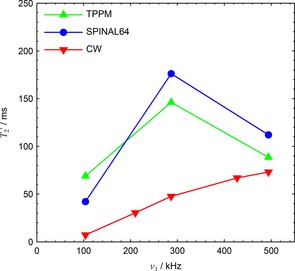
Peak experimental $T_{2}^{'}$ as a function of ^1^H nutation rate at $\nu_{r}=12\;\mathrm{kHz}$ and $\nu_{0}^{H}=400\;\mathrm{MHz}$ . Hardware configuration 8 was used with a CP contact time of 2 ms (see Table 1).

Figure 10 shows optimal experimental $T_{2}^{'}$ values as a function of nutation frequency for TPPM, SPINAL‐64 and CW. Above *v*_1_≃300 kHz, the peak $T_{2}^{'}$ values for the phase‐modulated sequences decrease sharply towards those of simple CW decoupling. Other experimental measurements using this probe have shown a similar drop in peak $T_{2}^{'}$ for both TPPM and SPINAL‐64 above *v*_1_≃250 kHz.^30^

**Figure 11:**
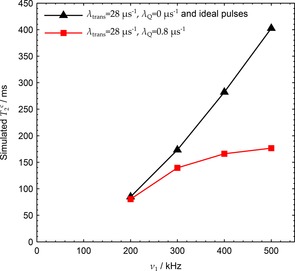
Peak simulated $T_{2}^{c}$ using a CH_6_ spin‐system at $\nu_{r}=12\;\mathrm{kHz}$ and $\nu_{0}^{H}=400\;\mathrm{MHz}$ under TPPM decoupling. Values are optima over a range of phase excursions $0^{\circ }\leq \phi \leq 51^{\circ }$ . Results from simulations with only amplitude transients ($\lambda_{\mathrm{trans}}=28\;{\mu s}^{-1},\lambda_{Q}=0\;{\mu s}^{-1}$ ) were negligibly different from those with no transients, and both are represented by black triangles. RF inhomogeneity was incorporated with 20 RF points using a nutation spectrum acquired on the same μMAS probe.

Simulations were performed with and without transients over a range of RF nutation frequencies. A value of $\lambda_{\mathrm{trans}}=\omega_{0}/2Q=28\;{\mu s}^{-1}$ is expected for the microcoil probe based on its Q 47, 70 (45 at 400 MHz for ^1^H). The value of $\lambda_{Q}$ depends on the exact probe tuning and, in lieu of precise measurements, the $\lambda_{Q}=0.8\;{\mu s}^{-1}$ value measured on the Bruker 2.5 mm probe was assumed to be representative. To find the optimum $T_{2}^{c}$ at a given nutation frequency, care was taken to ensure that the phase and pulse width simulation step sizes were small enough to show smooth evolution of dephasing times. Figure 11 demonstrates that the observed decrease in $T_{2}^{c}$ at $\nu_{1}\geq 200$ kHz is due to the combined effects of in‐phase and quadrature transients. The in‐phase transients have little impact on their own, at least for TPPM with $\nu_{1}\gg \nu_{r}$ . This agrees with our understanding that only a small reduction in dephasing time, owing to a minor reduction in the average RF amplitude, will be observed if the effective nutation axis of the sequence remains in the x-y plane. This is true in the presence of solely amplitude transients. If the nutation axis tilts out of the x-y plane as a result of the presence of both amplitude and quadrature transients, however, then a larger impact on dephasing times is expected, especially at high RF where decoupling optima are narrow.

## 3 Conclusions

Although heteronuclear decoupling has been widely investigated, the diversity of previous studies has made it difficult to make quantitative comparisons. The difficulty of optimising multi‐parameter decoupling sequences, particularly in regions where the decoupling optima are narrow, means that the results presented in the prior literature are often inconsistent. Measuring decoupling performance by using a well‐defined protocol and a wide variety of experimental conditions has provided large data sets that can be mined to address different questions. Here, we focus on understanding what are the limiting factors determining decoupling performance under different experimental conditions. Table 2 compiles the peak $T_{2}^{'}$ values observed here with previous literature results.^21^, ^27^

**Table 2:** Peak values of glycine C${}_{\alpha }$ $T_{2}^{'}$ observed under different experimental conditions.

$T_{2}^{'}$ [ms]^[a]^	$\nu_{0}^{H}$ [MHz]	MAS rate [kHz]	$\nu_{1}$ [kHz]	Sequence	Reference
61.1(5)	500	10	115	SW_*f*_‐TPPM‐sc	[21]
133(3)	500	25	170	TPPM	this work
82(1)	500	62.5	170	TPPM	this work
38(1)	850	12	105	TPPM	this work
101(4)	850	25	170	TPPM	this work
83.5(5)	850	62.5	170	TPPM	this work

[a] Figures in parentheses are one standard deviation uncertainties from fitting.

The decrease in decoupling performance with increasing magnetic field was expected for decoupling sequences, such as CW and TPPM, which are primarily limited by cross‐terms between the heteronuclear dipolar coupling and the ^1^H chemical shift anisotropy, but is less expected for sequences such as XiX decoupling (which is primarily limited by purely dipolar terms). This is likely to reflect higher‐order terms involving the ^1^H CSA and also offsets in ^1^H NMR frequencies from the decoupling frequency. In practice, the greater magnetisation losses at higher field will be largely offset by the intrinsically greater sensitivity and resolution of spectra obtained at higher field. Note that large ^1^H CSAs, such as those often observed in amide groups,^74^ are expected to have an analogous effect on decoupling performance to increasing the magnetic field.

As has previously been observed with ^13^C linewidths, decoupling performance measured by $T_{2}^{'}$ values is systematically worse at MAS rates above 60 kHz compared with 25 kHz. This reflects the increased number of “resonance” conditions in the area of parameter space where decoupling is typically optimal when the MAS rate is of the order of the ^1^H nutation frequency. “Low power” decoupling offers distinct advantages in these regimes; indeed, an impressive $T_{2}^{'}$ of 200 ms has been observed on a similar test sample (glycine ethyl ester) using a modified form of XiX decoupling at 90 kHz MAS.^75^ Resonance conditions are less significant in the low spinning speed regime, and $T_{2}^{'}$ values *increase* with spinning rates below approximately 22 kHz MAS.^21^, ^27^

RF transients associated with phase switches are not found to have a significant impact at typical ^1^H nutation rates. At nutation rates above 300kHz , however, simulations and experiments show that phase transients have an increasing impact, particularly from their quadrature components. At nutation rates of 500 kHz or more, achievable in microcoils, the performance of phase‐modulated sequences decreases dramatically towards that of simple CW decoupling. These problems can be addressed by careful tune‐up to minimise quadrature transients and/or development of sequences that are robust with respect to transients.

Inhomogeneity of the radio‐frequency field has a significant impact on $T_{2}^{'}$ , essentially by “smoothing off” peak decoupling conditions. Probes with flatter homogeneity profiles produce $T_{2}^{'}$ decay curves that are closer to exponential and with measurably longer $T_{2}^{'}$ values. Such factors contribute to the difficulty of reproducing $T_{2}^{'}$ quantitatively in simulation,^13^ and introduce a probe‐to‐probe variation that reduces the transferability of optimal decoupling sequences and parameters. This may be particularly problematic at higher NMR frequencies, where λ/4 approaches the dimensions of the transmitter coil. The option of restricting the sample to regions with a more uniform RF profile is relatively unattractive, as improvements in sensitivity owing to longer $T_{2}^{'}$ will be more than offset by the overall loss of signal. Coil geometries with flatter RF profiles, such as the end‐compensated coils introduced by Yannoni and co‐workers,^76^ would be a better alternative, provided that overall RF performance can be maintained. There may also be greater scope for optimising probes at higher field; the noticeably poorer RF inhomogeneity of the probe used at 850 MHz (Figure S4 in the Supporting Information) will have had some impact on the $T_{2}^{'}$ values obtained.

The peak values of the time constant for nuclear spin decay, $T_{2}^{'}$ , of C${}_{\alpha }$ in glycine summarised in Table 2 provide useful “reference” points when setting up experiments involving refocussing. By measuring the signal intensity after the refocussing period as a function of the decoupling parameters, the decoupling can be readily optimised, in a similar fashion to the optimisation of decoupling during acquisition periods. Indeed, $T_{2}^{'}$ can be readily estimated from the reduction of signal intensity relative to a reference experiment without the refocussing period, as used here to acquire parameter maps efficiently. These reference values can be used to judge the scope for further optimisation.

Although the best results were mostly obtained here using straightforward two‐pulse phase‐modulated (TPPM) decoupling, “good enough” decoupling for a given application may be achievable by using more easily optimised sequences. For example, r CW^20^ and variants^17^, ^18^, ^19^ may get close to these values through single‐parameter optimisations, whereas modifications of TPPM, such as SW_*f*_‐TPPM^64^ and its supercycled variant SW_*f*_‐TPPM‐sc,^77^ are more tolerant to parameter mis‐set. Where optimal performance is critical, for example, to measure very small couplings, the detailed parameter maps obtained here, such as Figure S9 (in the Supporting Information), should be invaluable guides to efficient optimisation. The high values obtained have very real experimental significance for experiments involving small couplings; a $T_{2}^{'}$ of 100 ms allows couplings as small as a few Hz to be measured and means that challenging correlation experiments, such as refocussed INADEQUATE, are viable, even at natural isotopic abundance.

## Supporting information

As a service to our authors and readers, this journal provides supporting information supplied by the authors. Such materials are peer reviewed and may be re‐organized for online delivery, but are not copy‐edited or typeset. Technical support issues arising from supporting information (other than missing files) should be addressed to the authors.

Supplementary
