# Supplementary material for: Rationalising Heteronuclear Decoupling in Refocussing Applications of Solid‐State NMR Spectroscopy
Source: Chemphyschem. 2017 Jan 23;18(4):394–405. doi: 10.1002/cphc.201601003 (PMC5396389; doi:10.1002/cphc.201601003)
Supplement: Supplementary file 1 — Supplementary [file CPHC-18-394-s001.pdf]

CHEMPHYSCHEM

Supporting Information

Rationalising Heteronuclear Decoupling in Refocussing Applications of Solid-State NMR Spectroscopy

Ilya Frantsuzov,[a] Suresh K. Vasa,[b] Matthias Ernst,[c] Steven P. Brown,[d] Vadim Zorin,[e, f] Arno P. M. Kentgens,[b] and Paul Hodgkinson*[a]

cphc_201601003_sm_miscellaneous_information.pdf

1 Parameter Map Scaling

The quick, but approximate, technique of measuring experimental T_2' values for a range of decoupling sequence parameters described in Section 2.1 tends to give less accurate measurements of T_2' when the decay is much faster or slower compared with the 2τ of the second measured point—compare dashed line and black points in Figure S1(a). This can be partly compensated by measuring several full T_2' decays under a range of decoupling conditions and comparing these exact T_2' values with the approximate values as shown in Figure S1(b). The relationship between the two sets of measurements was found to be reasonably well described by a simple polynomial, which was then used to rescale the approximate T_2' values (solid line in Figure S1(a)) to better reflect the exact T_2' measurements.

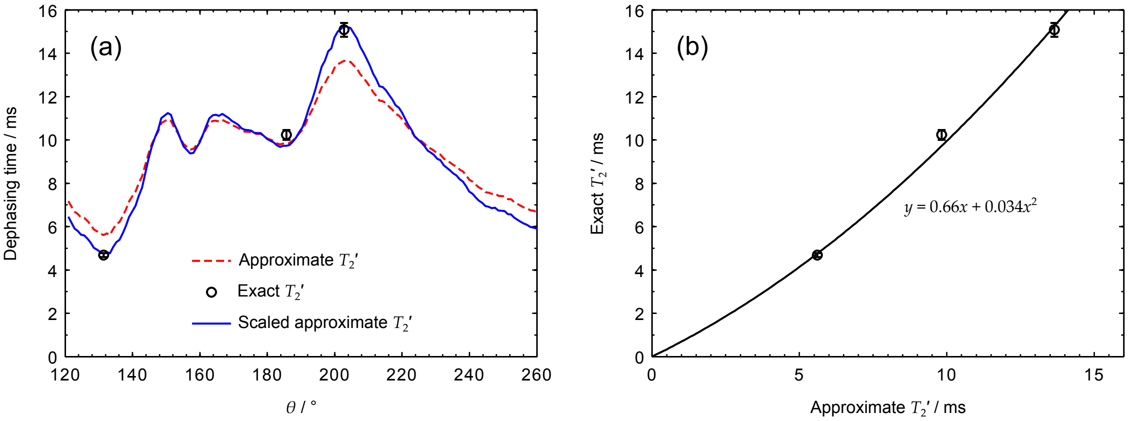

Figure S1: (a) Cross-section through the experimental SPINAL-64 [1] parameter map across a range of pulse widths at $\phi = 6°$, $\nu_r = 12$ kHz, $\nu_1 = 105$ kHz and $\nu_0^{\mathrm{H}} = 600$ MHz: T_2' estimated from pairs of points at $2\tau = 0, 15$ ms (red-dash), exact T_2' measured from full decays (black circles) and adjusted T_2' based on scaling of initial estimates (solid blue line). (b) Polynomial function fit describing mapping of estimated T_2' values at points where full decays were measured and used to scale the approximate T_2' values.

2 PISSARRO-5 Decoupling

Figure S2 demonstrates the difference between T_2^* and T_2' parameter maps under XiX [2,3] and PISSARRO-5 [4] decoupling. T_2^* was measured as $1/(\pi \times \mathrm{FWHM})$ of the tallest spectral peak, and then this value was scaled down for the remaining map points in proportion to their peak heights.

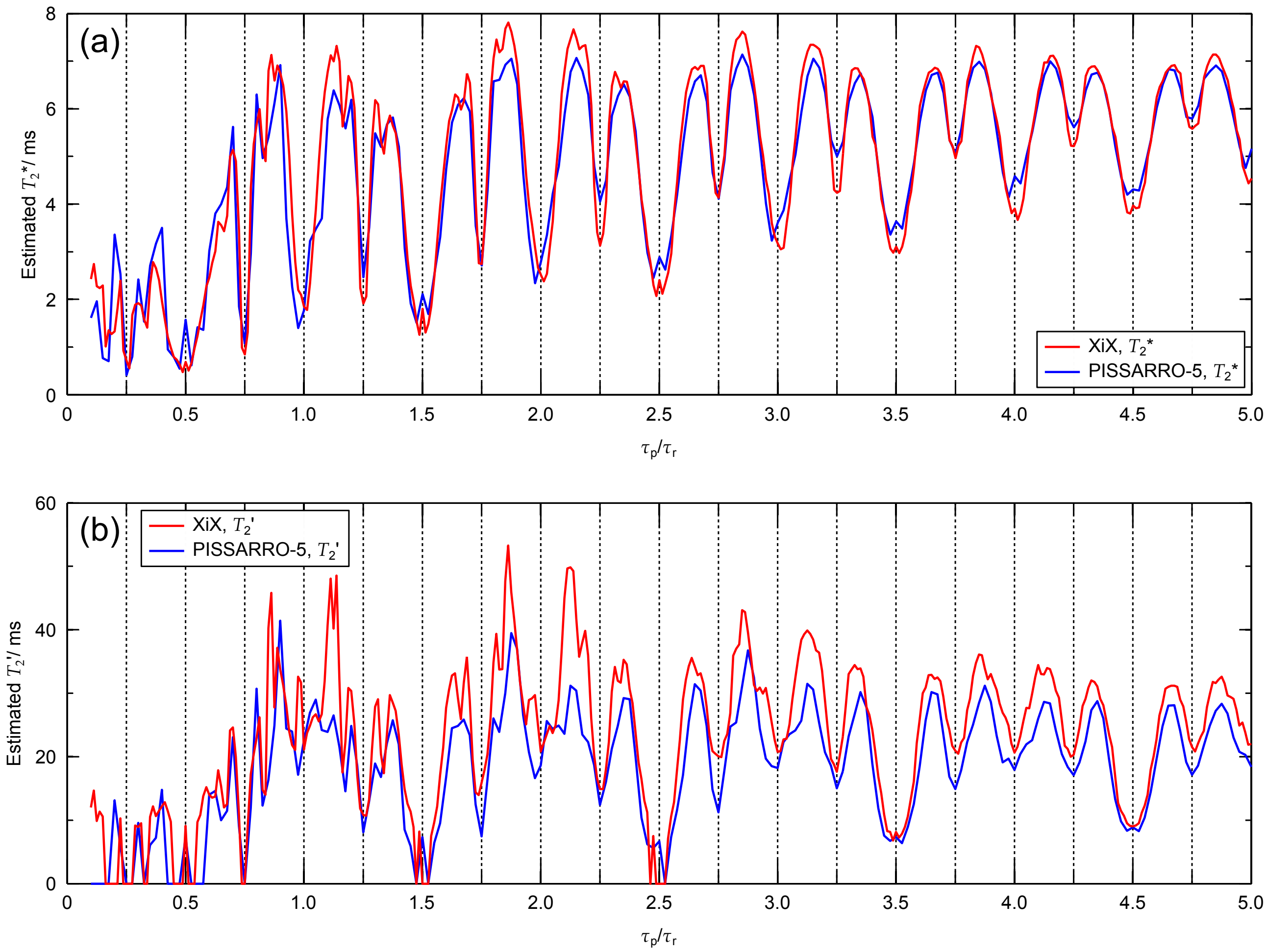

Figure S2: (a) T_2^* and (b) T_2' parameter maps of (red) XiX and (blue) PISSARRO-5, both using $\nu_r = 25$ kHz, $\nu_1 = 170$ kHz at $\nu_0^{\mathrm{H}} = 500$ MHz. For XiX the CP contact time was 1.2 ms and the pulse width increment was 0.5 μs. For PISSARRO-5 the CP contact time was 0.85 ms and the pulse width increment was 1 μs. Hardware configuration 2 was used (see Table 1). The vertical dashed lines represent homonuclear and heteronuclear recoupling resonance conditions as described in Ref. [5].

3 Simulation Details

Text of this subsection reproduced from Ref. [6]. Spin-systems containing different numbers of protons at increasing distance from a selected methylene C atom were created, based on the room temperature neutron structure of α-glycine (CSD refcode GLYCIN20 [7]). These spin systems are labelled as CH_n, with n indicating the number of protons in the system. CASTEP version 6.0 [8] was used to optimise the ^{1}H positions in the unit cell using a planewave cut-off energy of 600 eV. Brillouin zone integrals used a minimum sampling density of 0.1 Å^{-1} apart with the sampling grid offset by 0.25, 0.25, 0.25 in fractional coordinates of the reciprocal lattice. The exchange-correlation functional was approximated at the generalised-gradient level, specifically that of Perdew, Burke and Ernzerhof (PBE) [9]. Ultrasoft pseudopotentials [10] consistent with the PBE approximation were generated by CASTEP on-the-fly. Shielding tensors were subsequently calculated using the GIPAW method [11–13].

The effects of dynamics on the dipolar and shielding tensors of protons of the NH_3^+ groups, which are in rapid exchange at ambient temperature, were accounted for by averaging the chemical shift and dipolar coupling tensors over the three ^{1}H positions and diagonalising to obtain the new principal components and mean tensor orientation. Dipolar coupling tensors between the spins of the NH_3^+ were reconstructed by re-orienting the averaged dipolar tensor along the C–NH_3^+ bond vector and scaling by $P_2(\cos 90°) = 1/2$. This task of combining shielding tensor information from CASTEP and dipolar couplings determined from the geometry was handled with in-house software (available with the pNMRsim simulation program [14]). The dynamics of ^{1}H coupled networks are strongly determined by the root-sum-square of the ^{1}H dipolar couplings, d_{rss}, at a given site [15], and so the contributions of neglected protons outside the extracted 'cluster' of spins to d_{rss} were compensated for by scaling the ^{1}H homonuclear dipolar couplings so that the d_{rss} at one of the methylene ^{1}H sites (H5 in GLYCIN20) of the reduced spin-system matched that of the extended lattice. This d_{rss} value converges to 27.8 kHz when sufficient unit cells are considered (the value for the other methylene proton, H4, is very similar, 27.3 kHz). Note that d_{rss} for H5 without motional averaging is 30.2 kHz. The heteronuclear dipolar couplings were not scaled since the heteronuclear couplings between C_α and non-methylene protons have a negligible effect on the heteronuclear d_{rss} values. The ^{1}H chemical shift referencing was chosen to bring the methylene protons on resonance by subtracting the calculated chemical shielding values from 26.56 ppm. The ^{13}C chemical shift and the negligible J couplings were not included in the spin systems. The resulting ^{13}C,$(^1$H$)_n$ spin systems are given the labels CH_n in the text.

Simulations of RF decoupling under magic-angle spinning were performed in Hilbert space with pNMRsim [14], using a minimum time-step for propagator calculation of 1 μs. The theoretical background to such simulations has been extensively described elsewhere [16–19]. The simulations started with a state of ^{13}C x magnetisation and measured the remaining x magnetisation as a function of the duration of the decoupling period to create a simulated free-induction decay (FID) or spin-echo decay. In spin-echo simulations, an ideal refocusing π-pulse [20] was applied at the mid-point of the rotation-synchronised decay time. Unless

otherwise indicated, powder averages were performed over all three Euler angles describing the crystallite orientation, using 150 orientations distributed over a hemisphere generated with the ZCW algorithm [21–23]. Where the cycle times of the RF pulse sequence and sample spinning are not too dissimilar, it is generally possible to find a common time base for both the timing of the RF pulse sequence and MAS period. For phase-modulated RF pulse sequences, this allows the evolution of the density matrix to be determined from a limited number of propagators evaluated over a single period of rotation [24], greatly reducing the simulation time, usually by an order of magnitude or more. T_2 relaxation can be safely omitted from these simulations by noting that at room temperature the relaxation of the C_α site of glycine is in the extreme narrowing limit where $T_1 = T_{1\rho} = T_2$; relaxation time constants on the order of seconds have been observed experimentally [25], much longer than the maximum T_2' observed for this site [26]. Although the ^{1}H T_1 is somewhat shorter (about 0.5–1 second), this is also orders of magnitude longer than the time constants for decay of the ^{1}H magnetisation due to "spin diffusion". The fast dynamics of the methyl group is helpful in shortening ^{1}H T_1 without contributing significantly to ^{1}H $T_{1\rho}$ [27]. When comparing time constants for coherent decay from simulation, T_2^{c}, with experimental T_2' values, it is important to take into account the inhomogeneity of the RF (B_1) field in typical NMR probes. The incorporation of RF inhomogeneity into the simulations is discussed below.

4 Simulating RF Inhomogeneity

Text adapted from section 5 of the Supplementary Information of Ref. [6], which also describes an alternative, more approximate, method to including RF inhomogeneity than the "exact" method described here.

Due to the shape of the RF coil (usually a solenoid), the B_1 field experienced by the sample will be inhomogeneous—the largest variation typically being the axial inhomogeneity along the length of the rotor with the field being lower towards the rotor tips. This in turn leads to a distribution of nutation rates across the sample, which is detrimental to decoupling sequences that require careful calibration of pulse tip-angles, θ, such as TPPM. Measuring the nutation spectrum of a probe, as described in Section 2.1, effectively provides a histogram of nutation rates experienced by the sample, whose effects were incorporated into the simulations in one of two ways.

The first approach is to repeat the simulation several times, using different decoupling nutation rates, scaling the ideal RF (corresponding to the modal nutation frequency in the nutation spectrum) for different regions of the nutation spectrum. The different magnetisation decays over a range of nutation rates for a given θ are summed and fitted to a mono-exponential to give the inhomogeneity-broadened T_2^{c}. The integration is most efficient if each simulation contributes equally to the sum, i.e. if each simulation corresponds to the same sample volume. This is done by dividing the nutation spectrum into segments of equal integral, with the centre-of-mass of each segment giving the nutation frequency to be used, as illustrated in figure S3. In practice using 15–20 equally weighted simulations was

found to sample the nutation spectrum sufficiently to reproduce experimental behaviour, broadening out "resonance" conditions observed at individual values of the RF nutation rate to produce smooth cross-sections as a function of θ.

A quicker, but less robust approach to simulating RF inhomogeneity comes from considering changes in ν_1 as effectively changes in the pulse tip-angle, θ (a parameter that is normally varied anyway in the process of simulating a parameter map), which allows, in some circumstances, the effects of RF inhomogeneity to be included without doing additional simulations. This approach can be taken if a sufficiently large range of tip-angles are simulated, in excess of $\theta_{\mathrm{min}}/2 < \theta < 1.2\theta_{\mathrm{max}}$ around the region of interest $\theta_{\mathrm{min}} < \theta < \theta_{\mathrm{max}}$. Furthermore, the decoupling performance as a function of θ in the region of interest should not include any major resonance conditions that are not linearly dependent on ν_1, such as those encountered in TPPM between the pulse width and MAS spinning rate. If these criteria are met, then the inhomogeneity-broadened decay at each θ can be calculated as a weighted sum of neighbouring magnetisation decays. Firstly, because the synchronisation algorithm results in acquisition dwell times that depend on the value of θ across a parameter map, the decays were linearly interpolated to a common timebase, typically one rotor period, before summation. Once the weighted decays are summed, the result is down-sampled to the dwell-time corresponding to the peak nutation rate and fitted to a mono-exponential to give an inhomogeneity-broadened T_2^{c} at a given θ. The weighting factors are determined based on integrals of the nutation spectrum as a function of the simulated tip-angles. The results from this quick RF inhomogeneity calculation are in reasonable agreement with the exact calculation in the region of interest, $\theta_{\mathrm{min}} < \theta < \theta_{\mathrm{max}}$, and this approach is useful as a good first-order approximation for time-consuming many-spin simulations. For example, the exact inhomogeneity simulation results shown in Fig. 7(a) are comparable to those using the approximate method.

Figure S4 shows the nutation spectra for hardware configurations 1–4 (see Table 1), relevant to the data shown in subsection 3.2. The broadness of the nutation spectrum at $\nu_0^{\mathrm{H}} = 850$ MHz (hardware configuration 4) could be attributed to better matching between the ^{1}H and ^{13}C channels, or fewer turns on the coil necessitated by the higher B_0 field. Some nutation spectra exhibited negative dips, such as that seen in Fig. S4 for $\nu_0^{\mathrm{H}} = 850$ MHz. These were not due to truncation of the nutation oscillation and not consistent for a given hardware configuration. They are therefore likely to be related to the MAS sidebands observed in the nutation spectra, as discussed in subsection 3.3.

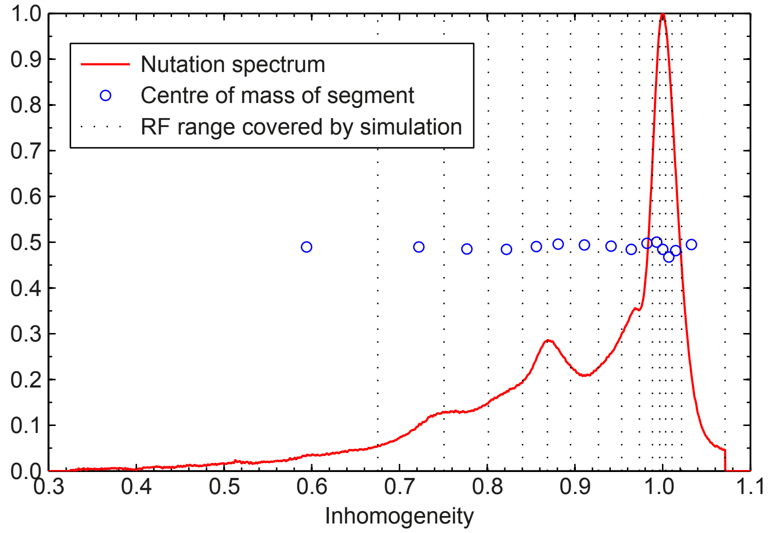

Figure S3: Illustration of the sampling of an experimental nutation profile in terms of 15 simulation points covering approximately equal areas of the nutation profile. The x-coordinate of the blue circles indicates the nutation frequency used for that simulation and corresponds to the centre-of-mass of that segment. The y-coordinates of the blue circles are the relative integrals of each segment and are used to weight the contribution of each FID to the sum.

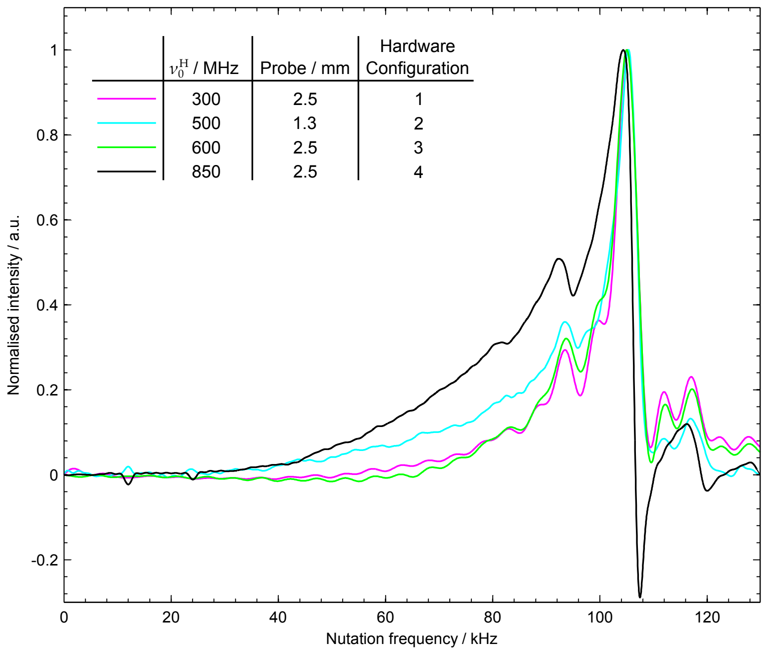

Figure S4: ^{1}H nutation spectra, measured via CP on ^{13}C at ν_r = 12 kHz. Hardware configurations 1–4 (see Table 1) were used, with CP contact times of 2.7, 1.2, 2.7, and 1.8 ms respectively.

5 Decoupling Transmitter Offset

Figure S5 compares decoupling performance as a function of ^{1}H transmitter offset for all the sequences studied across a range of experimental conditions.

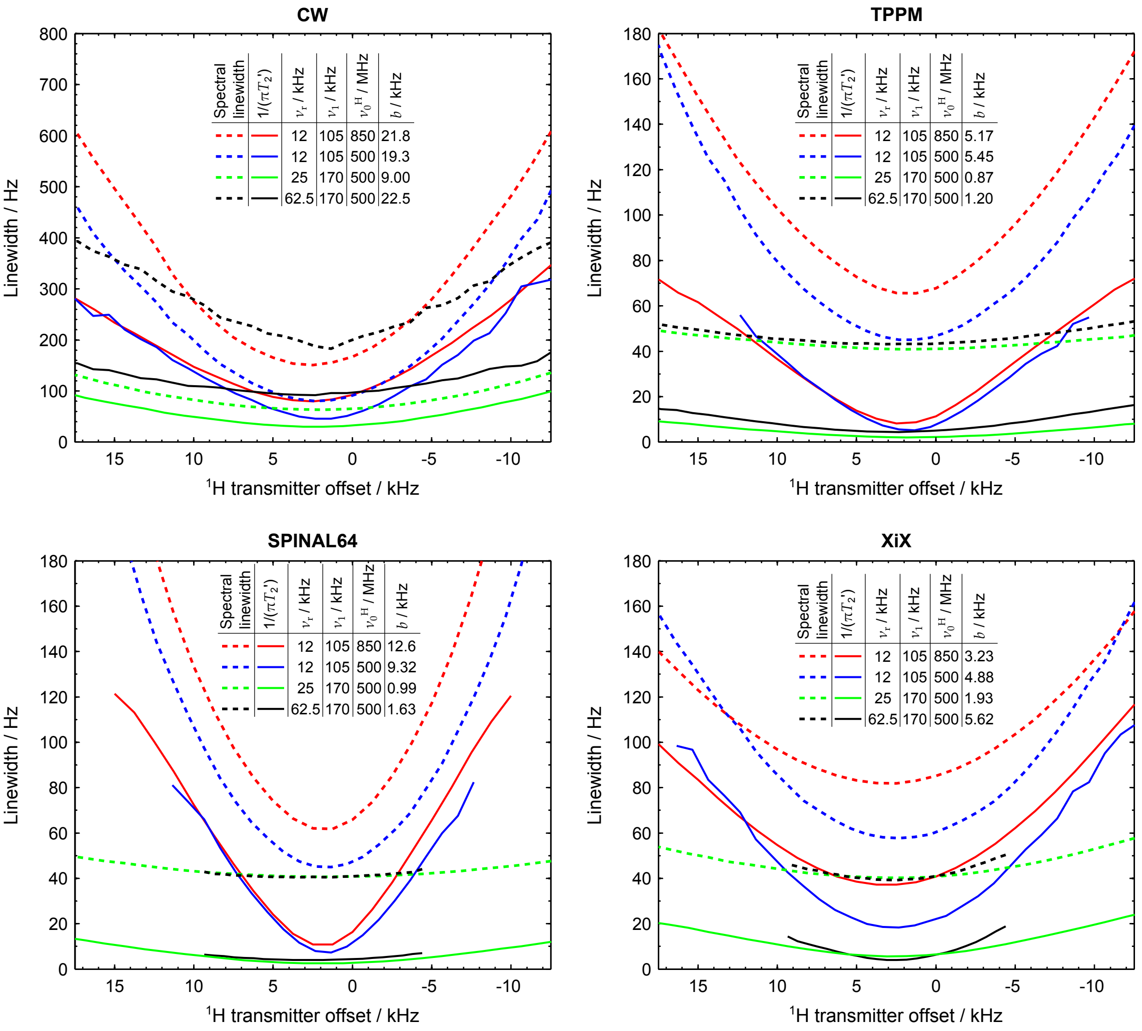

Figure S5: ^{1}H transmitter offset dependence of CW, TPPM, SPINAL-64, and XiX under various experimental conditions. Plots for TPPM and XiX are reproduced from Fig. 2 and shown here for ease of comparison. All sequence parameters were optimised across the range of offsets. The spectral linewidth was measured as the FWHM of the peak. For data at $\nu_0^{\mathrm{H}} = 850$ MHz, hardware configuration 4 was used with a CP contact time of 1.8 ms (see Table 1). For data at $\nu_0^{\mathrm{H}} = 500$ MHz, hardware configuration 2 was used with CP contact times of 1.2, 1.2 and 1.5 ms at $\nu_r = 12, 25$ and 62.5 kHz respectively. The b values refer to fits of the spectral linewidths as a function of transmitter offset, Δ, to $\mathrm{LW} = \mathrm{LW}_{\min} + b((\Delta - \Delta_{\min})/\nu_1)^2$.

6 B_0 and ν_r Dependence of T_2'

Collated results of the dependence of T_2' on B_0 under various optimised decoupling sequences are shown in Fig. S6 for slow MAS, and in Fig. S7 for moderate and fast MAS. Data from Table 1 of Ref. [28] is included for comparison. Note that the peak maximum of the unresolved ^{1}H spectrum is close to the NH_3^+ resonance in the case of glycine.

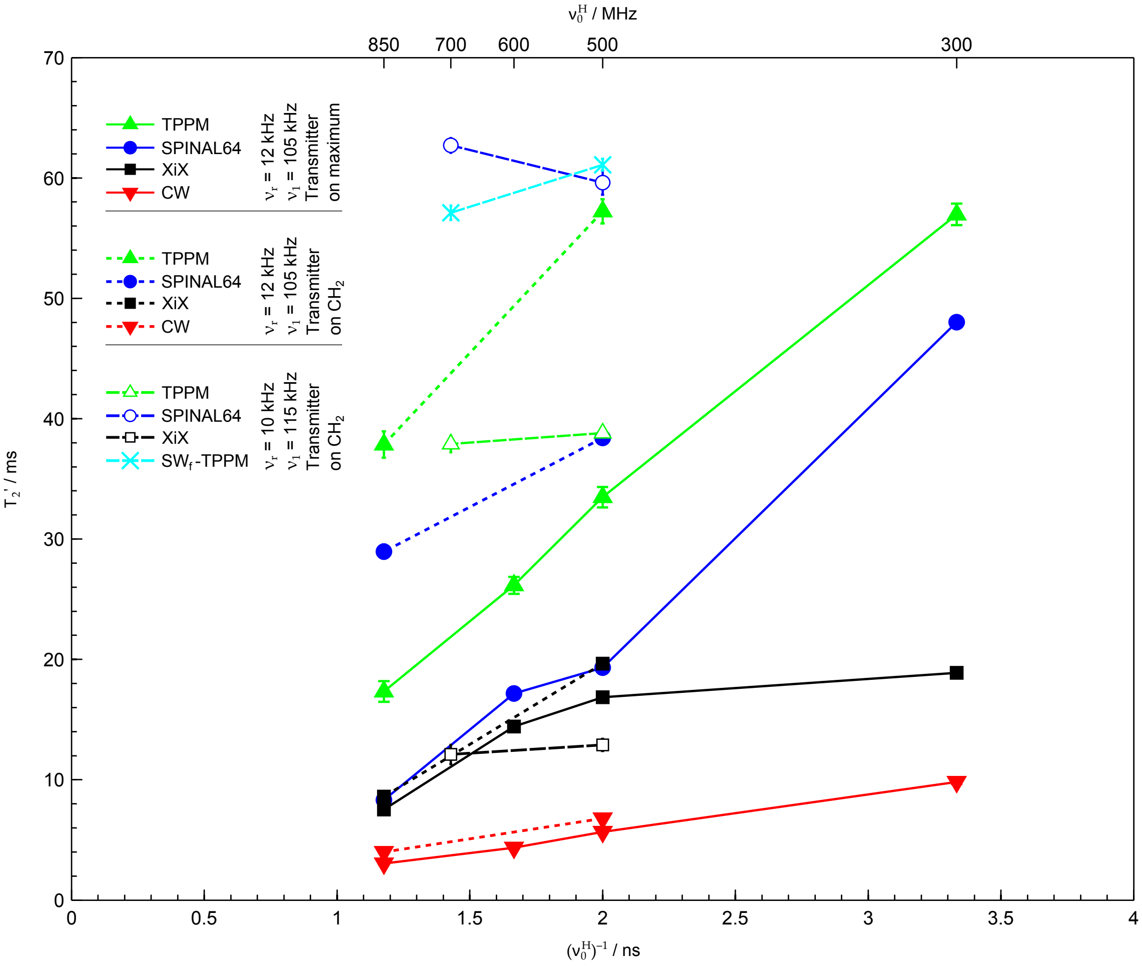

Figure S6: Experimental T_2' as a function of ν_0^{H} under optimised TPPM, SPINAL-64, XiX, CW and SW$_f$-TPPM [29] decoupling. (Solid lines and short-dash) Data acquired using $\nu_r = 12$ kHz and $\nu_1 = 105$ kHz (XiX used $\nu_r = 11.905$ kHz to ensure sychronisation of pulse width increments with the MAS period). Hardware configurations 1–4 were used, with CP contact times of 2.7, 1.2, 2.7 and 1.8 ms, respectively (see Table 1). (Long-dash) Data acquired under $\nu_r = 10$ kHz and $\nu_1 = 115$ kHz, transcribed from Table 1 of Ref. [28]. The ^{1}H transmitter offset used in each dataset is indicated in the legend. Note that the SPINAL phase parameters were separately optimised in Ref. [28] ($\phi = 8°$, $\alpha = 2°$, $\beta = 2\alpha$).

Figure S8 shows the results of Fig. S7 as a function of ν_r together with data from Fig. 8(a) of Ref. [30] for comparison. Note the significant improvement in T_2' going from $\nu_1 = 150$ to 170 kHz at $\nu_r = 25$ kHz. Such conditions of moderate MAS rates and high-power

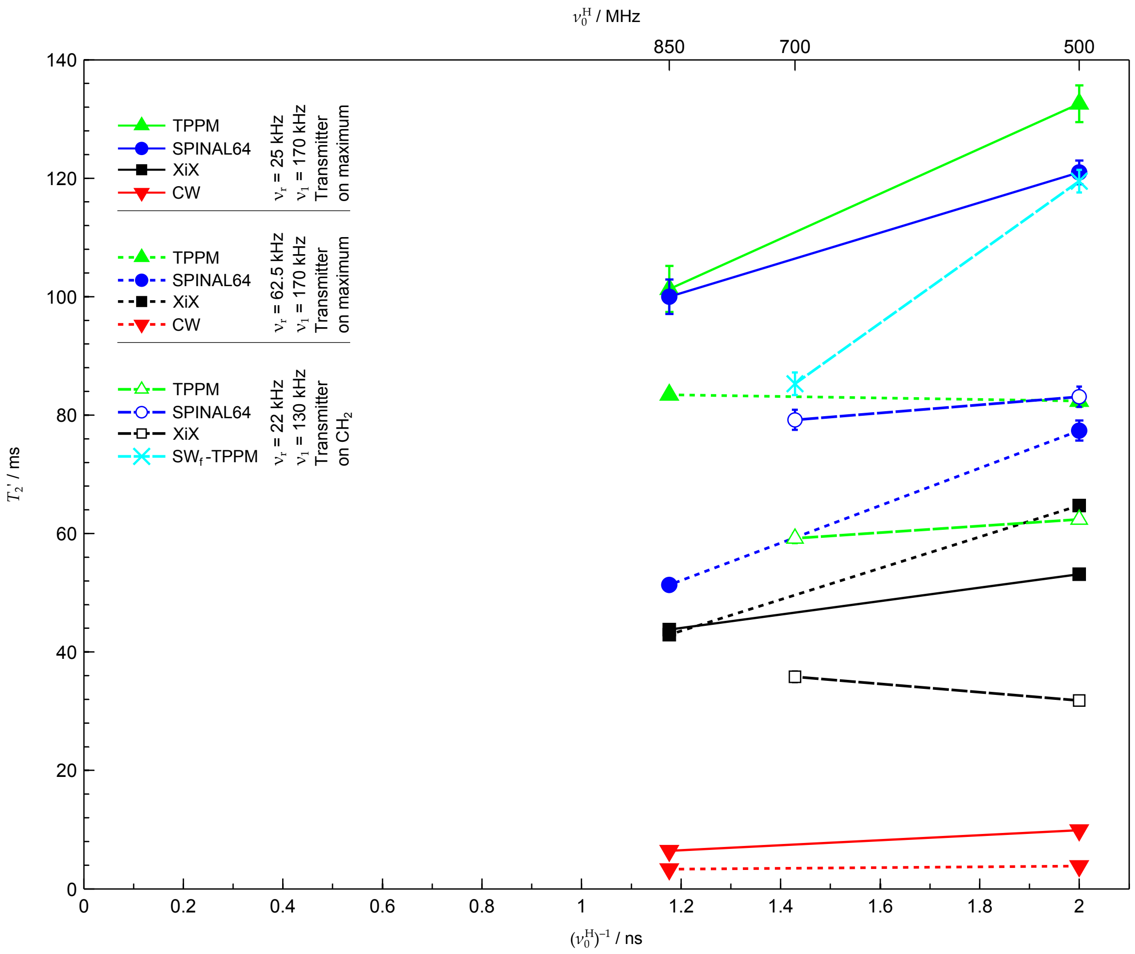

Figure S7: Experimental T_2' as a function of $\nu_0^{\rm H}$ under optimised TPPM, SPINAL-64, XiX, CW and SW$_f$-TPPM [29] decoupling. (Solid lines) Acquired under $\nu_r = 25$ kHz and $\nu_1 = 170$ kHz, using hardware configurations 2 and 5 (see Table 1) with CP contact times of 1.2 and 2.5 ms respectively. (Short-dash) Acquired under $\nu_r = 62.5$ kHz and $\nu_1 = 170$ kHz, using hardware configurations 2 and 5 with CP contact times of 1.5 and 1.2 ms respectively. (Long-dash) Data acquired under $\nu_r = 22$ kHz and $\nu_1 = 130$ kHz, transcribed from Table 1 of Ref. [28]. The ^{1}H transmitter offset used in each dataset is indicated in the legend. Note that the SPINAL phase parameters were separately optimised in Ref. [28] ($\phi = 8°$, $\alpha = 2°$, $\beta = 2\alpha$).

decoupling appear to be optimal for TPPM and SPINAL-64.

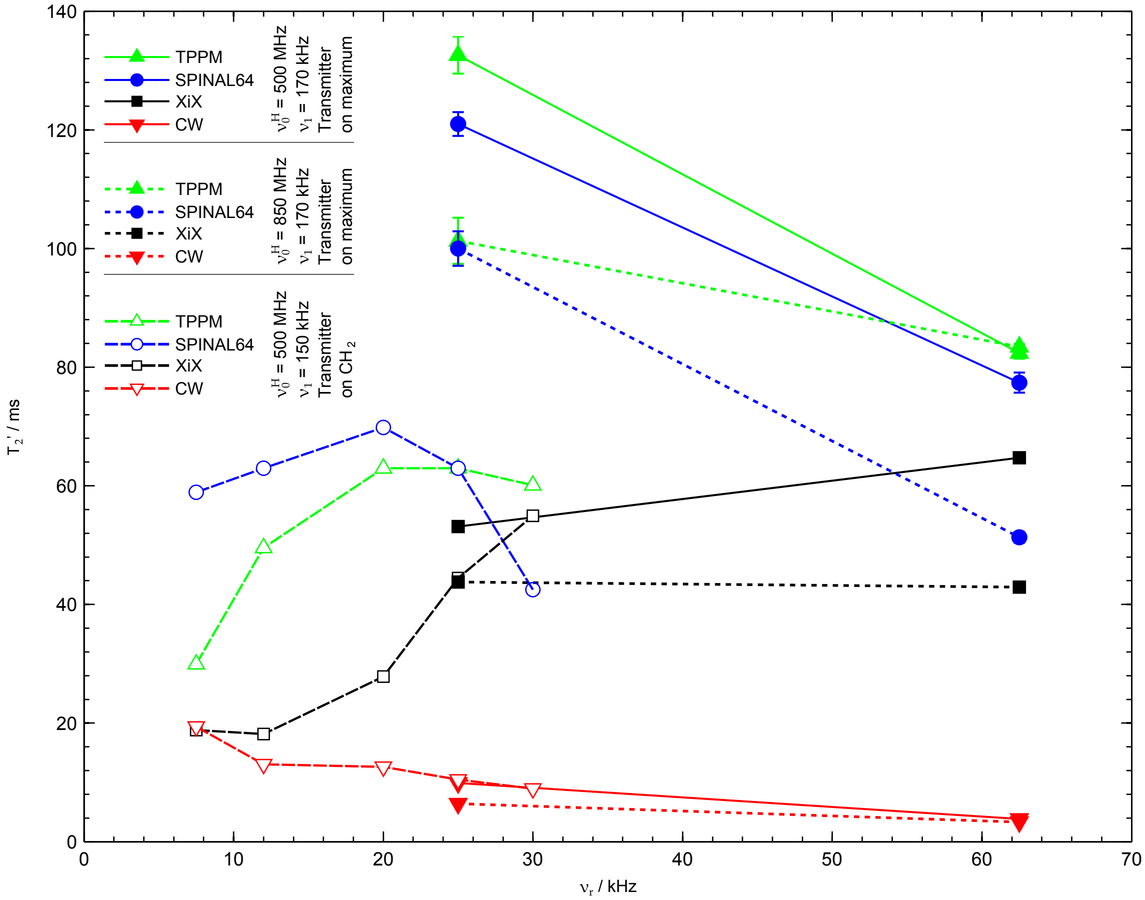

Figure S8: Experimental T_2' as a function of ν_r under optimised TPPM, SPINAL-64, XiX, and CW decoupling. (Solid lines) Acquired at $\nu_0^{\mathrm{H}} = 500$ MHz and $\nu_1 = 170$ kHz, using hardware configuration 2 (see Table 1) with CP contact times of 1.2 and 1.5 ms for $\nu_r = 25$ and 62.5 kHz respectively. (Short-dash) Acquired at $\nu_0^{\mathrm{H}} = 850$ MHz and $\nu_1 = 170$ kHz, using hardware configuration 5 with CP contact times of 2.5 and 1.2 ms for $\nu_r = 25$ and 62.5 kHz respectively. (Long-dash) Data acquired at $\nu_0^{\mathrm{H}} = 500$ MHz and $\nu_1 = 150$ kHz, taken from Figure 8(a) of Ref. [30]. The ^{1}H transmitter offset used in each dataset is indicated in the legend.

7 Sequence Parameter Maps

Note the T_2^* maps below use the calculated $T_2^* = 1/(\pi\mathrm{FWHM})$ from the highest peak in the data set as the reference, and this value is scaled down for the remaining map points in proportion to their peak heights.

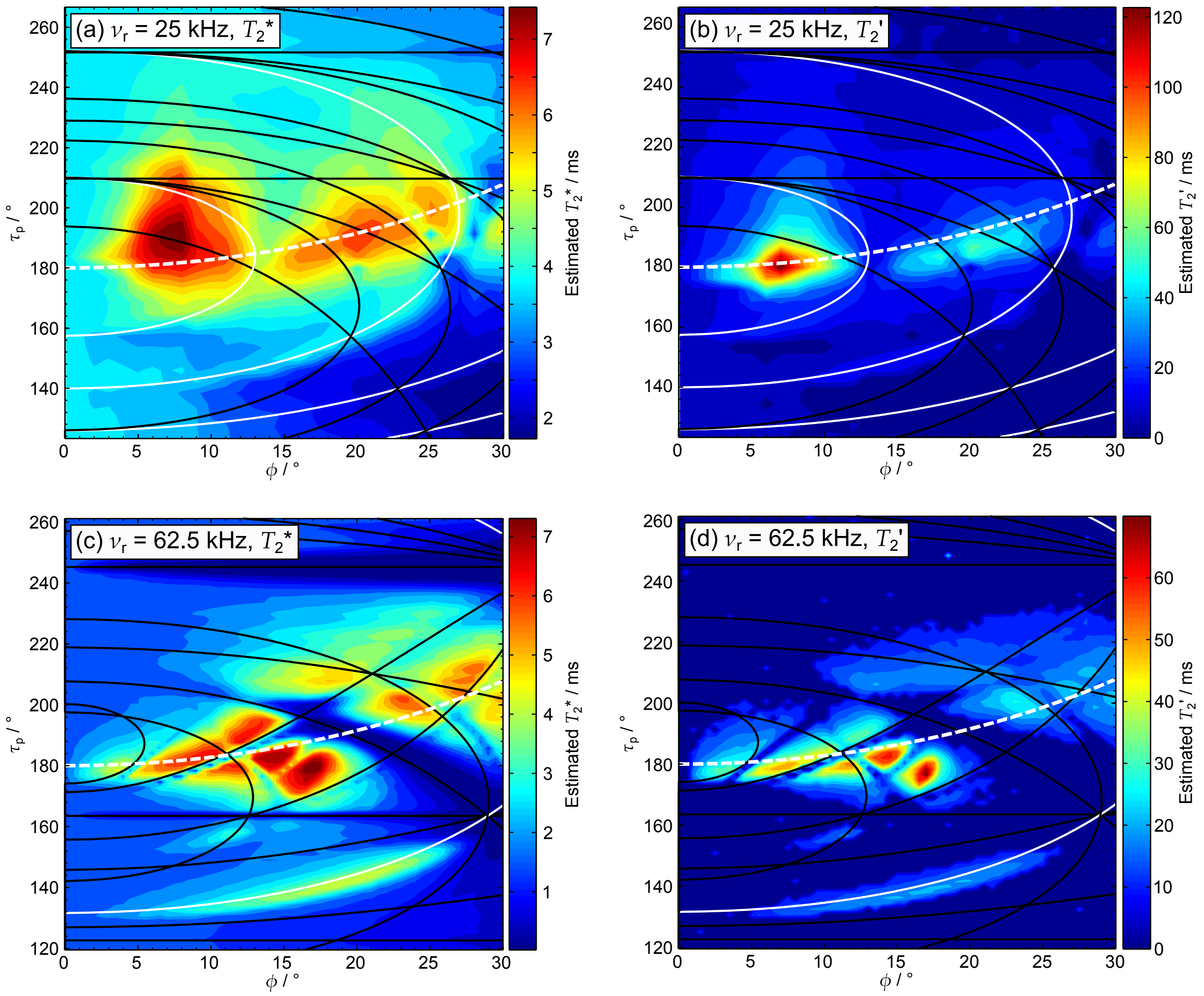

Figure S9: TPPM parameter maps of (a) T_2^* at $\nu_r = 25$ kHz, (b) T_2' at $\nu_r = 25$ kHz, (c) T_2^* at $\nu_r = 62.5$ kHz, (c) T_2' at $\nu_r = 62.5$ kHz, all using $\nu_1 = 170$ kHz at $\nu_0^{\mathrm{H}} = 500$ MHz. The solid lines represent recoupling resonance conditions for (black) heteronuclear interactions and (white) purely homonuclear interactions as described in [31]. The dashed white line, $\tau_p^{180}/\cos\phi$, shows where decoupling optima are expected to be found. For $\nu_r = 25$ kHz, the CP contact time was 1.2 ms, the pulse width increment was 0.06 μs and the phase increment was 1°. For $\nu_r = 62.5$ kHz, the CP contact time was 1.5 ms, the pulse width increment was 0.03 μs and the phase increment was 0.5°. Hardware configuration 2 was used (see Table 1).

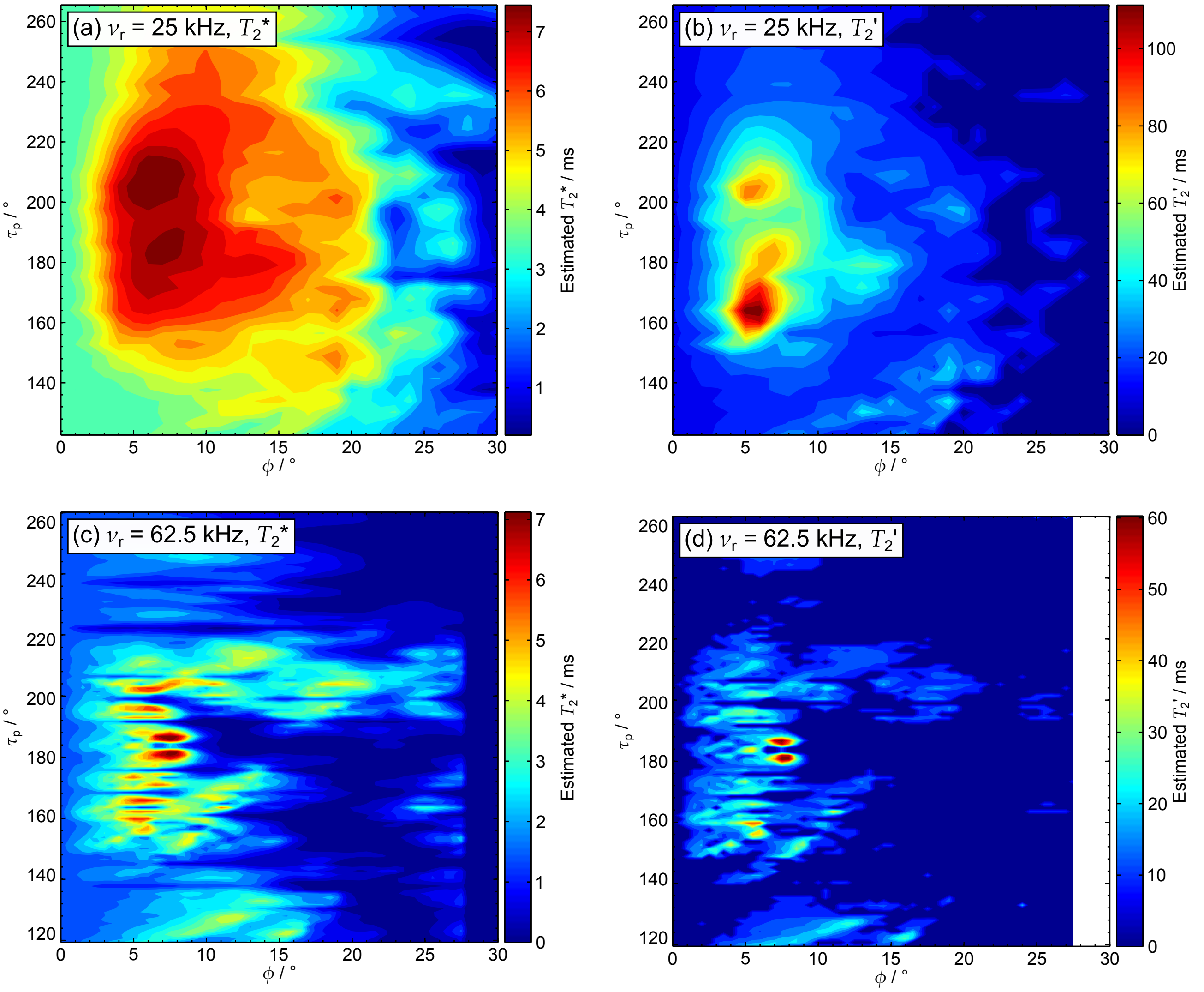

Figure S10: SPINAL-64 parameter maps of (a) T_2^* at $\nu_r = 25$ kHz, (b) T_2' at $\nu_r = 25$ kHz, (c) T_2^* at $\nu_r = 62.5$ kHz, (c) T_2' at $\nu_r = 62.5$ kHz, all using $\nu_1 = 170$ kHz at $\nu_0^{\mathrm{H}} = 500$ MHz. For $\nu_r = 25$ kHz, the CP contact time was 1.2 ms, the pulse width increment was 0.06 μs and the phase increment was 1°. For $\nu_r = 62.5$ kHz, the CP contact time was 1.5 ms, the pulse width increment was 0.02 μs and the phase increment was 0.5°. Hardware configuration 2 was used (see Table 1).

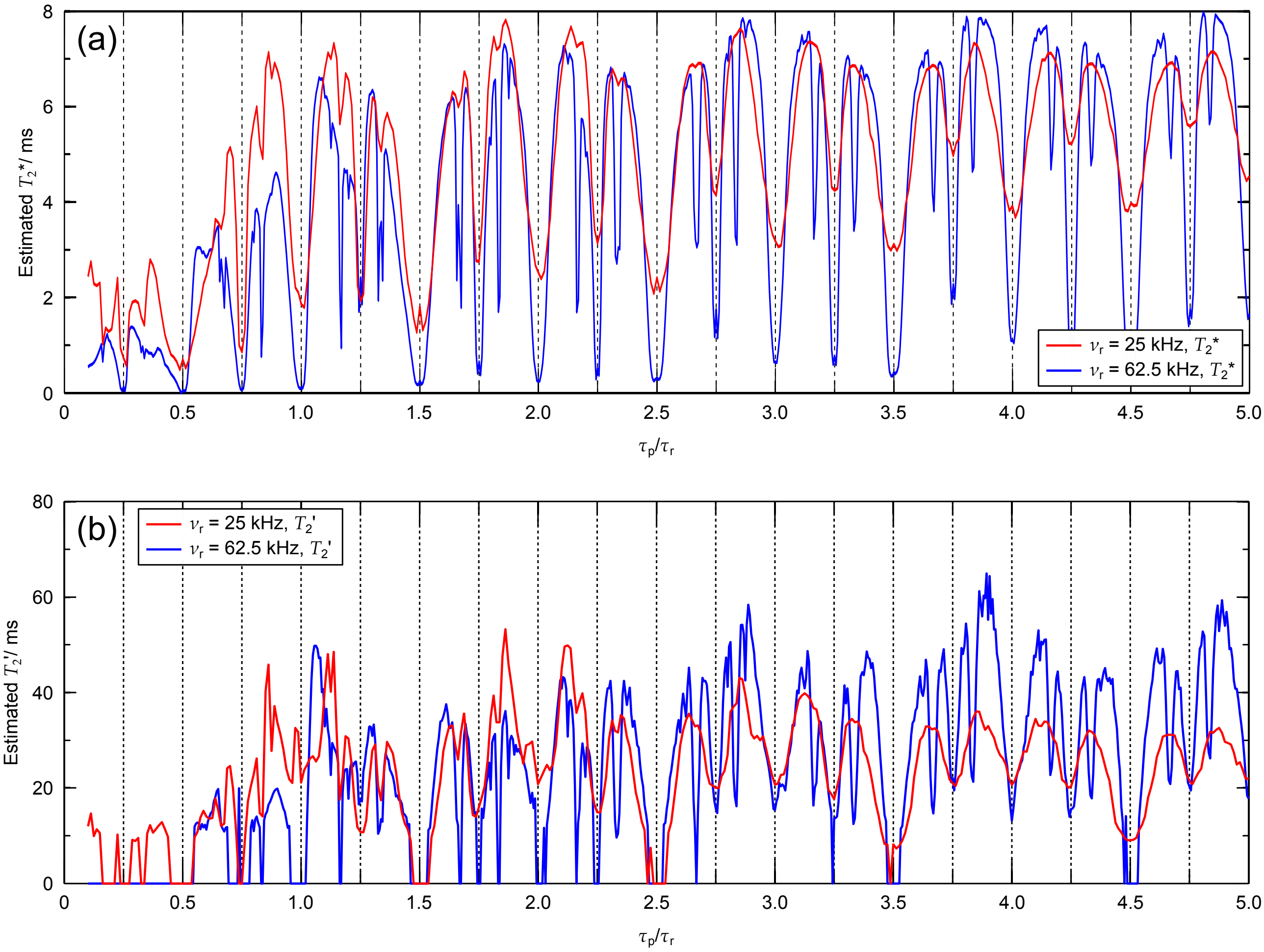

Figure S11: (a) T_2^* and (b) T_2' XiX parameter maps at (red) $\nu_r = 25$ kHz and (blue) $\nu_r = 62.5$ kHz, both using $\nu_1 = 170$ kHz at $\nu_0^{\mathrm{H}} = 500$ MHz. For $\nu_r = 25$ kHz, the CP contact time was 1.2 ms and the pulse width increment was 0.5 μs. For $\nu_r = 62.5$ kHz, the CP contact time was 1.5 ms and the pulse width increment was 0.1 μs. Hardware configuration 2 was used (see Table 1). The vertical dashed lines represent homonuclear and heteronuclear recoupling resonance conditions as described in Ref. [5].

Table S1: Summary of available datasets available through data DOI given in the main text

ν_0^{H}/ MHz	ν_r/ kHz	ν_1/ kHz	Sample	Probe	Parameter Maps
300	12	105	glycine-2-^{13}C,^{15}N	2.5 mm	TPPM 2D and 1D, SPINAL-64 2D and 1D, XiX
300	12	105	L-alanine-2-^{13}C,^{15}N	2.5 mm	TPPM 2D and 1D, SPINAL-64 2D and 1D, XiX
400	12	104	glycine-2-^{13}C,^{15}N	Microcoil MAS	TPPM 2D, SPINAL-64 1D, XiX
400	12	210.3	glycine-2-^{13}C,^{15}N	Microcoil MAS	CW
400	12	286.6	glycine-2-^{13}C,^{15}N	Microcoil MAS	TPPM 2D, SPINAL-64 1D, XiX
400	12	427.2	glycine-2-^{13}C,^{15}N	Microcoil MAS	CW
400	12	494.3	glycine-2-^{13}C,^{15}N	Microcoil MAS	TPPM 2D, SPINAL-64 1D, XiX
400	12	650	glycine-2-^{13}C,^{15}N	Microcoil MAS	CW
500	25	105	glycine-2-^{13}C,^{15}N	2.5 mm	TPPM 2D and 1D, SPINAL-64 2D and 1D, XiX
500	12	105	glycine-2-^{13}C,^{15}N	1.3 mm	TPPM 1D, SPINAL-64 1D, XiX
500	25	105	glycine-2-^{13}C,^{15}N	1.3 mm	TPPM 2D, SPINAL-64 2D and 1D, XiX, PISSARRO-5
500	25	170	glycine-2-^{13}C,^{15}N	1.3 mm	TPPM 2D and 1D, SPINAL-8 2D and 1D, SPINAL-16 2D and 1D, SPINAL-32 2D and 1D, SPINAL-64 2D and 1D, XiX, PISSARRO-5
500	62.5	170	glycine-2-^{13}C,^{15}N	1.3 mm	TPPM 2D and 1D, SPINAL-64 2D and 1D, XiX, PISSARRO-5
500	25	105	L-alanine-2-^{13}C,^{15}N	1.3 mm	TPPM 2D, SPINAL-64 2D and 1D, XiX
500	25	170	L-alanine-2-^{13}C,^{15}N	1.3 mm	TPPM 2D and 1D, SPINAL-64 2D, XiX
500	12	93	glycine-2-^{13}C,^{15}N	3.2 mm BioMAS	TPPM 2D and 1D, SPINAL-64 1D
500	12	93	glycine-2-^{13}C,^{15}N	3.2 mm T3	TPPM 2D and 1D, SPINAL-64 1D
600	25	105	glycine-2-^{13}C,^{15}N	1 mm	TPPM 2D, SPINAL-64 2D, XiX
600	25	347.7	glycine-2-^{13}C,^{15}N	1 mm	SPINAL-64 2D, XiX
600	78.125	213	glycine-2-^{13}C,^{15}N	1 mm	TPPM 2D, SPINAL-64 2D, XiX
600	12	105	glycine-2-^{13}C,^{15}N	2.5 mm	TPPM 2D and 1D, SPINAL-64 2D and 1D, XiX
850	12	105	glycine-2-^{13}C,^{15}N	2.5 mm	TPPM 2D and 1D, SPINAL-64 2D and 1D, XiX
850	25	170	glycine-2-^{13}C,^{15}N	1.3 mm	TPPM 1D, SPINAL-64 1D, XiX
850	62.5	170	glycine-2-^{13}C,^{15}N	1.3 mm	TPPM 2D and 1D, SPINAL-64 2D and 1D, XiX

References

[1] B. M. Fung, A. K. Khitrin, and K. Ermolaev, J. Magn. Reson. **142**, 97 (2000).

[2] P. Tekely, P. Palmas, and D. Canet, J. Magn. Reson. A **107**, 129 (1994).

[3] A. Detken, E. H. Hardy, M. Ernst, and B. H. Meier, Chem. Phys. Lett. **356**, 298 (2002).

[4] M. Weingarth, P. Tekely, and G. Bodenhausen, Chem. Phys. Lett. **466**, 247 (2008).

[5] M. Ernst, H. Geen, and B. H. Meier, Solid State Nucl. Magn. Reson. **29**, 2 (2006).

[6] I. Frantsuzov, M. Ernst, S. P. Brown, and P. Hodgkinson, Solid State Nucl. Magn. Reson. **70**, 28 (2015).

[7] P. Langan, S. A. Mason, D. Myles, and B. P. Schoenborn, Acta Crystallogr., Sect. B: Struct. Sci. **58**, 728 (Aug 2002).

[8] S. J. Clark, M. D. Segall, C. J. Pickard, P. J. Hasnip, M. I. J. Probert, K. Refson, and M. C. Payne, Z. Kristallogr. **220**, 567 (2005).

[9] J. P. Perdew, K. Burke, and M. Ernzerhof, Phys. Rev. Lett. **77**, 3865 (1996).

[10] D. Vanderbilt, Phys. Rev. B **41**, 7892 (1990).

[11] C. J. Pickard and F. Mauri, Phys. Rev. B **63**, 245101 (2001).

[12] R. K. Harris, P. Hodgkinson, C. J. Pickard, J. R. Yates, and V. Zorin, Magn. Reson. Chem. **45**, S174 (2007).

[13] C. Bonhomme, C. Gervais, F. Babonneau, C. Coelho, F. Pourpoint, T. Azaïs, S. E. Ashbrook, J. M. Griffin, J. R. Yates, F. Mauri, and C. J. Pickard, Chem. Rev. **112**, 5733 (2012).

[14] P. Hodgkinson, *pNMRsim: a general simulation program for large problems in solid-state NMR*, URL: http://www.durham.ac.uk/paul.hodgkinson/pNMRsim.

[15] V. E. Zorin, S. P. Brown, and P. Hodgkinson, Mol. Phys. **104**, 293 (2006).

[16] P. Hodgkinson and L. Emsley, Prog. Nucl. Magn. Reson. Spectrosc. **36**, 201 (2000).

[17] M. Bak, J. T. Rasmussen, and N. C. Nielsen, J. Magn. Reson. **147**, 296 (2000).

[18] M. Edén, Concept. Magnetic Res. A **17A**, 117 (2003).

[19] M. Veshtort and R. G. Griffin, J. Magn. Reson. **178**, 248 (2006).

[20] N. S. Barrow, J. R. Yates, S. A. Feller, D. Holland, S. E. Ashbrook, P. Hodgkinson, and S. P. Brown, Phys. Chem. Chem. Phys. **13**, 5778 (2011).

[21] S. K. Zaremba, Ann. Mat. Pur. Appl. **73**, 293 (1966).

[22] H. Conroy, J. Chem. Phys. **47**, 5307 (1967).

[23] V. B. Cheng, H. H. Suzukawa, Jr., and M. Wolfsberg, J. Chem. Phys. **59**, 3992 (1973).

[24] V. E. Zorin, M. Ernst, S. P. Brown, and P. Hodgkinson, J. Magn. Reson. **192**, 183 (2008).

[25] A. Krushelnitsky, R. Kurbanov, D. Reichert, G. Hempel, H. Schneider, and V. Fedotov, Solid State Nucl. Magn. Reson. **22**, 423 (2002).

[26] S. K. Vasa, H. Janssen, E. R. H. Van Eck, and A. P. M. Kentgens, Phys. Chem. Chem. Phys. **13**, 104 (2010).

[27] R. E. Taylor and C. Dybowski, J. Mol. Struct. **889**, 376 (2008).

[28] V. S. Mithu, S. Pratihar, S. Paul, and P. K. Madhu, J. Magn. Reson. **220**, 8 (2012).

[29] R. S. Thakur, N. D. Kurur, and P. K. Madhu, Chem. Phys. Lett. **426**, 459 (2006).

[30] G. De Paëpe, A. Lesage, and L. Emsley, J. Chem. Phys. **119**, 4833 (2003).

[31] I. Scholz, P. Hodgkinson, B. H. Meier, and M. Ernst, J. Chem. Phys. **130**, 114510 (2009).
